# Exploring novel soliton, wave solutions, and modulation instability analysis for the (3+1)-dimensional KP-SKR equation using the improved generalized Riccati equation mapping approach

**DOI:** 10.1038/s41598-025-22030-1

**Published:** 2025-10-29

**Authors:** Noha M. Kamel, Hamdy M. Ahmed, Wafaa B. Rabie

**Affiliations:** 1 Department of Physics and Engineering Mathematics, Faculty of engineering, Ain Shams University, Cairo, Egypt; 2 Department of Physics and Engineering Mathematics, Higher Institute of Engineering, El-Shorouk Academy, Cairo, Egypt; 3 Department of Mathematics, Faculty of Science, Luxor University, Taiba, Luxor Egypt

**Keywords:** Improved generalized Riccati equation mapping method (IGREMM), (3+1)-dimensional KP-SKR equation, Traveling wave solutions, Modulation instability analysis, Graphical representations, Mathematics and computing, Optics and photonics, Physics

## Abstract

In this work, new soliton and wave solutions for the (3+1)-dimensional Kadomtsev-Petviashvili-Sawada-Kotera-Ramani equation (KP-SKRE) were derived using the improved generalized Riccati equation mapping method (IGREMM). The KP-SKRE plays an essential role in modeling the phenomena of wave nonlinearity. The wave nonlinearity appears in fluid dynamics, plasma physics, and other fields that involve interactions between dispersion and nonlinearity. The obtained solutions include bright soliton, combo bright-dark soliton, singular soliton in addition to singular periodic, hyperbolic, exponential, and rational solutions. The obtained solutions describe different behaviors of wave propagation through nonlinear media and provide a deeper understanding of the dynamics of nonlinear waves governed by the KP-SKRE. The novelty of this work includes four key original contributions to the study of the KP-SKRE. First, the novel application of IGREMM to the KP-SKRE. Second, the derivation of previously unreported exact solutions, including combo bright-dark soliton and singular soliton, along with hyperbolic, exponential, rational, and singular periodic solutions. Third, the Bäcklund transformation of IGREMM can be used to construct additional novel forms of solution. Fourth, the conduction of the modulation instability (MI) analysis by the linear stability technique. The MI analysis reveals the conditions under which the wave solutions become unstable. The direct substitution of the derived solutions in the original equation confirmed their validity. For visualization, 2D, 3D, and density plots of some selected exact solutions were presented. This visualization illustrates the solution wave profile with the choice of parameters’ values that satisfy the constraints. Furthermore, graphical representations of the outcomes of MI analysis were provided to enhance the physical interpretation of the results. The findings of this study contribute to a better understanding of nonlinear wave dynamics. Potential applications of these findings can be found in oceanography, optical communications, and plasma physics where soliton and nonlinear wave solutions have significant importance.

## Introduction

The discovery of the solitary waves in shallow water made by Russell in 1844 was the keystone for the development of many nonlinear partial differential equations (NLPDEs) models. The shallow water surface waves with long wavelength and small amplitude were modeled by the Korteweg and de Vries (KdV) equation in 1895. Even though Korteweg and de Vries introduced the KdV equation in 1895, the study of solitary waves did not get scientists attention till 1965. This attention started after the discovery of the “soliton” by Zabusky and Kruskal in 1965. The soliton word was chosen to describe the behavior of solitary waves in a similar way to particles. Zabusky and Kruskal discovered that in a nonlinear crystal lattice the solitary waves, after collision, keep their speed and shape^1–6^. The development of new NLPDEs that model solitary waves and the search for their exact solutions are continuously growing ever since^7–16^.

The KdV equation was extended by Kadomtsev and Petviashvili in 1970 into two-dimensional form and was named as Kadomtsev–Petviashvili equation (KPE)^17^. Applying the inverse scattering transform to the KPE, soliton solutions were generated in^18^. KPE can model many physical phenomena in many fields such as water waves, nonlinear optics, and plasma physics. Another extension of the KdV equation was introduced by Sawada and Kotera in 1974 and was named as Sawada–Kotera equation (SKE)^19^. SKE was investigated in many researches, see^20–23^. A novel (2+1)-dimensional equation named Kadomtsev–Petviashvili– Sawada–Kotera–Ramani equation (KP-SKRE) was proposed in 2022^24^ which was the combination of the KPE and the SKE. The Lax integrability of KP-SKRE was also studied in^24^. In^25^, the Hirota’s bilinear method was applied to the (2+1)-dimensional KP-SKRE and lump solutions and interaction solutions were investigated.

### The governing model

The (2+1)-dimensional KP-SKRE was extended to the following $(3+1)$-dimensional KP-SKRE in^26^:

1 $$\begin{aligned} \begin{aligned}&\displaystyle Q_{xt}+\left( 3\, Q^2+Q_{xx}\right) _{xx} +\displaystyle \left( 15\,Q^3+15\,Q\,Q_{xx}\,+Q_{xxxx}\right) _{xx}+\alpha _1\,Q_{yy}+\alpha _2\,Q_{zz}\\&\quad \quad +\beta _1\, Q_{xy}+ \beta _2\,Q_{yz}+\beta _3\,Q_{xz}=0, \end{aligned} \end{aligned}$$

where $\alpha _1,$ and $\,\alpha _2$ are the coefficients of transverse dispersion in the $y-$direction, and $z-$direction, respectively. The values of the coefficients $\alpha _{1}$, and $\alpha _{2}$ specify the strength of dispersion in the $y-$direction, and $z-$direction, respectively. While, $\beta _1,$ $\beta _2,$ and $\beta _3$ are the coefficients of the interaction effects between different spatial dimensions. The values of the coefficients $\beta _{1},\,$ $\beta _{2}$, and $\beta _{3}$ control the effect of any variation in one spatial direction on the wave’s behavior in another spatial direction. Eq.(1) governs many physical systems, in which waves are long in one spatial direction relative to other spatial directions, and have one temporal direction. The behavior of these waves is affected by both the primary direction of propagation, and the variations in the transverse directions. The primary application of Eq.(1) is in the modeling of shallow water waves. But it is used in other applications like, for example, plasma physics where it describes the wave propagation in plasma, which is a matter of ions and electrons. Another example of applications is oceanography, where it describes ocean waves and is especially important to study rogue waves, and many other applications as nonlinear optics, fluid mechanics, and electrical engineering^26^. $Q(t,\,x,\,y,\,z)$ in Eq.(1) is a function in three spatial directions $x,\,y,\,$ and *z*, and one temporal direction which is time *t*. The function $Q(t,\,x,\,y,\,z)$ describes the free-surface elevation of shallow water waves in a dispersive medium, measured from a flat bottom. These waves have long wave lengths compared to the water depth, and are influenced by both the primary direction of propagation *x*, and the alternations in the transverse directions $y,\,$ and *z*.

This research investigated the $(3+1)$-dimensional KP-SKRE in Eq.(1) by the improved generalized Riccati equation mapping method (IGREMM) to find new analytic solutions. The IGREMM has great potential in the solution of different types of NLPDEs analytically. The advantages of IGREMM are easy application, straight forward calculations, and various types of exact solutions that can be generated by symbolic computation^27^. These advantages encouraged the application of IGREMM to different types of NLPDEs available in the literature. For example, in^28^, IGREMM was applied to solve the (2+1) dimensional Boiti–Leon–Pempinelle equation along with the extended tanh-function method. While, in^29^, IGREMM was combined with tanh–coth method to derive solutions for the nonlinear coupled equation in mathematical physics. Also, in^30^ IGREMM was applied to find exact solutions to the (3+1)-dimensional Jimbo–Miwa equation. In^31^, exact solutions for the discrete nonlinear electrical transmission lines in (2+1) dimensions were found by IGREMM. And many other researches are available in the literature^32–34^.

The main motivation of this work is to narrow the research gap in the literature considering the KP-SKRE in Eq.(1) by exploring new analytic solutions using IGREMM. Even though Eq.(1) is extremely important in many applications such as oceanography, nonlinear optics, fluid mechanics, and electrical engineering^26^, few research papers are available in literature. This work explores novel previously unreported analytic solutions by the novel application of IGREMM to Eq.(1). Among these analytic solutions are combo bright-dark soliton and singular soliton, along with hyperbolic, exponential, rational, and singular periodic solutions. The Bäcklund transformation of IGREMM provides the ability of construction of additional novel forms of solution. The modulation instability (MI) analysis by the linear stability technique for Eq.(1) was conducted in this study.

### Comparison with earlier work

According to our knowledge of previous work in the literature, the $(3+1)$-dimensional KP-SKRE in Eq.(1) was proposed in^26^ and multiple-soliton, and lump solutions were investigated by simplified Hirota’s scheme. In^35^, only one form of solution as a rational function in exponential was investigated for the $(3+1)$-dimensional KP-SKRE by the Kudryashov method. In^36^, the $(3+1)$-dimensional KP-SKRE was investigated by the Jacobi elliptic function expansion generating single and combined Jacobi elliptic function solutions. In^37^, hybrid solutions were investigated for the $(3+1)$-dimensional KP-SKRE by the Hirota bilinear method. This current work aims to add abundant novel analytic solutions for the $(3+1)$-dimensional KP-SKRE in Eq.(1) using the improved generalized Riccati equation mapping method.

The novelty of current work includes four key original contributions to the study of Eq.(1). First, the novel application of the improved generalized Riccati equation mapping method (IGREMM) to Eq.(1). Second, the derivation of previously unreported exact solutions, including combo bright-dark soliton and singular soliton, along with hyperbolic, exponential, rational, and singular periodic solutions. Third, the Bäcklund transformation of IGREMM can be used to construct additional novel forms of solution. Fourth, the conduction of the modulation instability (MI) analysis by the linear stability technique for Eq.(1). All derived solutions have been rigorously verified through direct substitution into the original equation.

The paper is arranged as follows: Sect. 2 details the IGREMM methodology. Section 3 presents its implementation and the obtained solutions. Section 4 develops the MI analysis. Section 5 provides graphical visualizations of the dynamics of some selected solutions through 2D, 3D and density plots, along with MI results. Section 6 concludes with a summary of findings and their implications.

## Improved generalized Riccati equation mapping method framework

This section presents the computational framework of the IGREMM^38,39^ for deriving exact solutions to NLPDEs. Consider the general NLPDE:

2 $$\begin{aligned} H (s,\, s_{x},\, s_{y},\ s_{z},\,s_{t},\,s_{xx},\, s_{yy},\,s_{zz},\,s_{tt},...)=0, \end{aligned}$$

where $s(t,\,x,\,y,\,z)$ is the wave solution of Eq.(2), the following steps are applied:

1. The application of the following wave transformation

3 $$\begin{aligned} \begin{array}{lcl} s(t,\,x,\,y,\,z) =F(\gamma ),\,\,\,\, \gamma =a_1\, x+a_2\, y+a_3\, z+a_4\, t, \end{array} \end{aligned}$$

to Eq.(2) reduces it to:

4 $$\begin{aligned} H(F,\,F',\,F'',\,F''',\,...)=0, \end{aligned}$$

*H* is a function in $F,\,F'=\displaystyle \frac{\textrm{d} F}{\textrm{d}\,\gamma },\,F''=\displaystyle \frac{\textrm{d}^{2}F}{\textrm{d}\,\gamma ^{2}},...etc.$

2. The solution to Eq.(4) is:

5 $$\begin{aligned} F(\gamma )=\displaystyle \sum _{j=0}^{m}\eta _{j}\,\Theta ^{j}(\gamma ), \,\,\,\,\displaystyle \eta _{m}\ne 0, \end{aligned}$$

where $$\Theta (\gamma )$$ satisfies:

6 $$\begin{aligned} \Theta ^{'}(\gamma )=\epsilon _{0}+\epsilon _{1}\,\Theta (\gamma )+\epsilon _{2}\,\Theta ^{2}(\gamma ), \end{aligned}$$

where *m* is an integer, $\eta _{j}\,(j=0,\,1,...,m)$, $\epsilon _{0},\, \epsilon _{1}$ and $\,\epsilon _{2}$ are parameters to be determined.

3 The integer *m* is determined by the balance between nonlinearity and dispersion of Eq.(4).
4 Eq.(4) turns into a polynomial in $\Theta (\gamma )$ after inserting Eqs.(5)-(6) in it.
5 The coefficients of the obtained polynomial in $\Theta (\gamma )$ are forced to vanish generating a system of equations in $\eta _{j}\,(j=0,\,1,...,m)$, $\epsilon _{0},\, \epsilon _{1}$, $\,\epsilon _{2}$, $\, a_1,\, a_2,\, a_3$ and $a_4$.
6 A suitable software, like Mathematica or Maple, is used to solve the obtained system of equations.
7 The exact solutions of Eq.(6) are listed in the following, with $\varepsilon$ as any arbitrary constant, and $b_{1}$ and $b_{2}$ are two arbitrary parameters:

I Hyperbolic solutions for $K=\epsilon _1^2-4\,\epsilon _0\,\epsilon _2>0$,   $\epsilon _0\,\epsilon _2\ne 0$,  or  $\epsilon _1\,\epsilon _2 \ne 0$, $b_1\ne 0$, and $b_2\ne 0$:

7 $$\begin{aligned} \displaystyle \Theta _{1}(\gamma )= & - \displaystyle \frac{\sqrt{K}\,\textrm{tanh}\left( \frac{\sqrt{K}\left( \varepsilon +\gamma \right) }{2}\right) }{2\,\epsilon _2}-\frac{\epsilon _{1}}{2\,\epsilon _{2}}, \end{aligned}$$

8 $$\begin{aligned} \displaystyle \Theta _{2}(\gamma )= & - \displaystyle \frac{\sqrt{K}\,\textrm{coth}\left( \frac{\sqrt{K}\left( \varepsilon +\gamma \right) }{2}\right) }{2\,\epsilon _2}-\frac{\epsilon _{1}}{2\,\epsilon _{2}}, \end{aligned}$$

9 $$\begin{aligned} \displaystyle \Theta _{3}^{\pm }(\gamma )= & - \displaystyle \frac{\sqrt{K}\,\left( \textrm{tanh}\left( \sqrt{K}\left( \varepsilon +\gamma \right) \right) \pm \textrm{i} \,\textrm{sech}\left( \sqrt{K}\left( \varepsilon +\gamma \right) \right) \right) }{2\,\epsilon _2}-\frac{\epsilon _{1}}{2\,\epsilon _{2}}, \end{aligned}$$

10 $$\begin{aligned} \displaystyle \Theta _{4}(\gamma )= & - \displaystyle \frac{\sqrt{K}\,\left( \textrm{tanh}\left( \frac{\sqrt{K}\left( \varepsilon +\gamma \right) }{4}\right) + \,\textrm{coth}\left( \frac{\sqrt{K}\left( \varepsilon +\gamma \right) }{4}\right) \right) }{4\,\epsilon _2}-\frac{\epsilon _{1}}{2\,\epsilon _{2}}, \end{aligned}$$

11 $$\begin{aligned} \displaystyle \Theta _{5}^{\pm }(\gamma )= & \displaystyle \frac{\pm \,\sqrt{K\left( b_1^2+b_2^2\right) }-\sqrt{K}\,b_1\,\textrm{cosh}\left( \sqrt{K}\left( \varepsilon +\gamma \right) \right) }{2\,\epsilon _2\,\left( b_1\,\textrm{sinh}\left( \sqrt{K}\,\left( \varepsilon +\gamma \right) \right) +b_2\,\right) }-\frac{\epsilon _{1}}{2\,\epsilon _{2}}, \end{aligned}$$

12 $$\begin{aligned} \displaystyle \Theta _{6}^{\pm }(\gamma )= & \displaystyle \frac{2\,\epsilon _0\textrm{cosh}\left( \sqrt{K}\left( \varepsilon +\gamma \right) \right) }{\sqrt{K}\,\textrm{sinh}\left( \sqrt{K}\left( \varepsilon +\gamma \right) \right) -\epsilon _1\,\textrm{cosh}\left( \sqrt{K}\left( \varepsilon +\gamma \right) \right) \pm \,\textrm{i}\,\sqrt{K}}, \end{aligned}$$

13 $$\begin{aligned} \displaystyle \Theta _{7}^{\pm }(\gamma )= & \displaystyle \frac{2\,\epsilon _0\textrm{sinh}\left( \sqrt{K}\left( \varepsilon +\gamma \right) \right) }{\sqrt{K}\,\textrm{cosh}\left( \sqrt{K}\left( \varepsilon +\gamma \right) \right) -\epsilon _1\,\textrm{sinh}\left( \sqrt{K}\left( \varepsilon +\gamma \right) \right) \pm \,\sqrt{K}}. \end{aligned}$$

II Trigonometric solutions for $K=\epsilon _1^2-4\,\epsilon _0\,\epsilon _2<0$,   $\epsilon _0\,\epsilon _2\ne 0$,  or  $\epsilon _1\,\epsilon _2 \ne 0$, and $b_1^2-b_2^2>0$:

14 $$\begin{aligned} \displaystyle \Theta _{8}(\gamma )= & \displaystyle \frac{\sqrt{-K}\,\textrm{tan}\left( \frac{\sqrt{-K}\left( \varepsilon +\gamma \right) }{2}\right) }{2\,\epsilon _2}-\frac{\epsilon _{1}}{2\,\epsilon _{2}}, \end{aligned}$$

15 $$\begin{aligned} \displaystyle \Theta _{9}(\gamma )= & - \displaystyle \frac{\sqrt{-K}\,\textrm{cot}\left( \frac{\sqrt{-K}\left( \varepsilon +\gamma \right) }{2}\right) }{2\,\epsilon _2}-\frac{\epsilon _{1}}{2\,\epsilon _{2}}, \end{aligned}$$

16 $$\begin{aligned} \displaystyle \Theta _{10}^{\pm }(\gamma )= & \displaystyle \frac{\sqrt{-K}\,\left( \textrm{tan}\left( \sqrt{-K}\left( \varepsilon +\gamma \right) \right) \pm \textrm{sec}\left( \sqrt{-K}\left( \varepsilon +\gamma \right) \right) \right) }{2\,\epsilon _2}-\frac{\epsilon _{1}}{2\,\epsilon _{2}}, \end{aligned}$$

17 $$\begin{aligned} \displaystyle \Theta _{11}(\gamma )= & \displaystyle \frac{\sqrt{-K}\,\left( \textrm{tan}\left( \frac{\sqrt{-K}\left( \varepsilon +\gamma \right) }{4}\right) - \,\textrm{cot}\left( \frac{\sqrt{-K}\left( \varepsilon +\gamma \right) }{4}\right) \right) }{4\,\epsilon _2}-\frac{\epsilon _{1}}{2\,\epsilon _{2}}, \end{aligned}$$

18 $$\begin{aligned} \displaystyle \Theta _{12}^{\pm }(\gamma )= & \displaystyle \frac{\pm \,\sqrt{-K\left( b_1^2-b_2^2\right) }-\sqrt{-K}\,b_1\,\textrm{cos}\left( \sqrt{-K}\left( \varepsilon +\gamma \right) \right) }{2\,\epsilon _2\,\left( b_1\,\textrm{sin}\left( \sqrt{-K}\,\left( \varepsilon +\gamma \right) \right) +b_2\,\right) }-\frac{\epsilon _{1}}{2\,\epsilon _{2}}, \end{aligned}$$

19 $$\begin{aligned} \displaystyle \Theta _{13}^{\pm }(\gamma )= & \displaystyle \frac{-2\,\epsilon _0\textrm{cos}\left( \sqrt{-K}\left( \varepsilon +\gamma \right) \right) }{\sqrt{-K}\,\textrm{sin}\left( \sqrt{-K}\left( \varepsilon +\gamma \right) \right) +\epsilon _1\,\textrm{cos}\left( \sqrt{-K}\left( \varepsilon +\gamma \right) \right) \pm \,\sqrt{-K}}, \end{aligned}$$

20 $$\begin{aligned} \displaystyle \Theta _{14}^{\pm }(\gamma )= & \displaystyle \frac{-2\,\epsilon _0\textrm{sin}\left( \sqrt{-K}\left( \varepsilon +\gamma \right) \right) }{\epsilon _1\,\textrm{sin}\left( \sqrt{-K}\left( \varepsilon +\gamma \right) \right) -\sqrt{-K}\,\textrm{cos}\left( \sqrt{-K}\left( \varepsilon +\gamma \right) \right) \pm \,\sqrt{-K}}. \end{aligned}$$

III Exponential solutions with $\lambda ,$ and *v* as arbitrary constants:
  1. For $$\epsilon _0=\lambda \, \epsilon _1$$, and $$\epsilon _2=0$$:
    21 $$\begin{aligned} \displaystyle \Theta _{15}(\gamma )= \displaystyle \textrm{e}^{\epsilon _{1}\,\left( \varepsilon +\gamma \right) }-\lambda . \end{aligned}$$
  2. For $$\epsilon _0=0,\,\epsilon _1\ne 0$$, and $$\epsilon _2\ne 0$$:
    22 $$\begin{aligned} \displaystyle \Theta _{16}(\gamma )= & -\displaystyle \,\frac{ \epsilon _1\,\nu }{\epsilon _{2}\,\displaystyle \left( \textrm{e}^{ \,-\epsilon _1\,\left( \varepsilon +\gamma \right) }+\nu \right) }, \end{aligned}$$
    23 $$\begin{aligned} \displaystyle \Theta _{17}(\gamma )= & -\displaystyle \,\frac{ \epsilon _1\,\textrm{e}^{ \,\epsilon _1\,\left( \varepsilon +\gamma \right) }}{\epsilon _{2}\,\displaystyle \left( \textrm{e}^{ \,\epsilon _1\,\left( \varepsilon +\gamma \right) }+\nu \right) }. \end{aligned}$$
    Rational solutions:
  3. For $$\epsilon _0=\epsilon _{1}=0$$,  and $$\,\epsilon _{2}\ne 0$$:
    24 $$\begin{aligned} \displaystyle \Theta _{18}^{\pm }(\gamma )= \pm \,\displaystyle \,\frac{1}{ \epsilon _{2}\left( \varepsilon +\gamma \right) }. \end{aligned}$$
  4. For $$\epsilon _0\ne 0,\,\epsilon _{1}\ne 0$$,  and $$\,\epsilon _{2}=\frac{\epsilon _1^2}{4\,\epsilon _0}$$:
    25 $$\begin{aligned} \displaystyle \Theta _{19}(\gamma )= - \,\displaystyle \,\frac{2\,\epsilon _0\left( \epsilon _1\left( \varepsilon +\gamma \right) +2\right) }{ \epsilon _{1}^{2}\left( \varepsilon +\gamma \right) }. \end{aligned}$$

8. The derived solutions for Eq.(2) are then obtained by substituting different forms of $\Theta (\gamma )$ into Eq.(5) then back into Eq.(3).
9. The Bäcklund transformation of IGREMM:

The Bäcklund transformation of Eq.(6) is given by:

26 $$\begin{aligned} \begin{array}{lcl} \displaystyle \Theta (\gamma )= \,\displaystyle \,\frac{-\epsilon _0\,D_1\,+\epsilon _2\,D_2\,\Theta _n (\gamma )}{ \epsilon _{1}\,D_1+\epsilon _2\,\left( D_2+\,D_1\,\Theta _n(\gamma )\right) }, \end{array} \end{aligned}$$

where $\Theta _n,\,n=1,...,19$ are defined in Eqs.(7)-(25), $D_1,\,D_2$ are arbitrary constants. By the use of Bäcklund transformation in Eq.(26), many other forms of exact solutions for Eq.(2) can be constructed. These described steps of IGREMM will be implemented on Eq.(1) in the next section.

## Application of IGREMM

### Methodology

This section presents the detailed implementation of IGEMM, described in section 2, to the (3+1)-dimensional Lax integrable KP-SKRE in Eq.(1).

1. Assume the analytic solution of Eq.(1) as:

27 $$\begin{aligned} Q(t,\,x,\,y,\,z)=F(\gamma ),\,\, \,\, \gamma =a_1\, x+a_2\, y+a_3\, z+a_4\, t. \end{aligned}$$

The insertion of Eq.(27) in Eq.(1) reduces it to:

28 $$\begin{aligned} \begin{aligned}&\left( \alpha _1 a_2^2+a_3^2 \alpha _2+a_3 a_2 \beta _2+a_1 \left( a_2 \beta _1+a_3 \beta _3+a_4\right) \right) F''(\gamma ) \\&\quad \quad +3 a_1^2 \left( F(\gamma ) (15 F(\gamma )+2) F''(\gamma )+(30 F(\gamma )+2) F'(\gamma )^2\right) \\&\quad \quad +a_1^4 \left( (15 F(\gamma )+1) F^{(4)}(\gamma )+15 F''(\gamma )^2+30 F^{(3)}(\gamma ) F'(\gamma )\right) +a_1^6 F^{(6)}(\gamma )=0. \end{aligned} \end{aligned}$$

2. Assume $$F(\gamma )$$ as:

29 $$\begin{aligned} F(\gamma )=\displaystyle \sum _{j=0}^{m}\eta _{j}\,\Theta ^{j}(\gamma ), \,\,\,\,\eta _{m}\ne 0. \end{aligned}$$

3. The balancing of dispersion and nonlinearity in E.(28) implies that $m=2$. Hence:

30 $$\begin{aligned} \displaystyle F(\gamma )=\eta _{0}+\eta _{1}\, \Theta (\gamma )+\,\eta _2\,\Theta ^2(\gamma ),\,\,\eta _2\ne 0. \end{aligned}$$

4. The substitution of Eqs.(30) and (6) into Eq.(28) generated a polynomial in $\Theta (\gamma ).$
5. The coefficients of $\Theta (\gamma )$ in the generated polynomial were set to zero. This produced the following system of equations:

$$\begin{aligned} & \displaystyle \underline{\Theta ^{0}\, coeff:}\\ & \quad \quad \epsilon _0 (\left( \eta _1 \epsilon _1+2 \eta _2 \epsilon _0\right) \left( \alpha _1 a_2^2+a_3^2 \alpha _2+a_3 a_2 \beta _2\right) +a_1 \left( a_2 \beta _1+a_3 \beta _3+a_4\right) \left( \eta _1 \epsilon _1+2 \eta _2 \epsilon _0\right) \\ & \quad \quad +a_1^6 \left( \eta _1 \epsilon _1 \left( \epsilon _1^4+52 \epsilon _0 \epsilon _2 \epsilon _1^2+136 \epsilon _0^2 \epsilon _2^2\right) +2 \eta _2 \epsilon _0 \left( 31 \epsilon _1^4+292 \epsilon _0 \epsilon _2 \epsilon _1^2+136 \epsilon _0^2 \epsilon _2^2\right) \right) \\ & \quad \quad +a_1^4 (60 \eta _2^2 \epsilon _0^3+2 \eta _2 \epsilon _0 \left( \left( 15 \eta _0+1\right) \left( 7 \epsilon _1^2+8 \epsilon _0 \epsilon _2\right) +120 \eta _1 \epsilon _0 \epsilon _1\right) +\eta _1 (\left( 15 \eta _0+1\right) \epsilon _1 \left( \epsilon _1^2+8 \epsilon _0 \epsilon _2\right) \\ & \quad \quad +15 \eta _1 \epsilon _0 \left( 3 \epsilon _1^2+4 \epsilon _0 \epsilon _2\right) ))+3 a_1^2 \left( \eta _0 \left( 15 \eta _0+2\right) \eta _1 \epsilon _1+2 \left( \left( 15 \eta _0+1\right) \eta _1^2+\eta _0 \left( 15 \eta _0+2\right) \eta _2\right) \epsilon _0\right) )=0,\\ & \displaystyle \underline{\Theta ^{1} coeff:}\\ & \quad \left( \eta _1 \left( \epsilon _1^2+2 \epsilon _0 \epsilon _2\right) +6 \eta _2 \epsilon _0 \epsilon _1\right) \left( \alpha _1 a_2^2+a_3^2 \alpha _2+a_3 a_2 \beta _2\right) +a_1 \left( a_2 \beta _1+a_3 \beta _3+a_4\right) (\eta _1 \left( \epsilon _1^2+2 \epsilon _0 \epsilon _2\right) \\ & \quad \quad +6 \eta _2 \epsilon _0 \epsilon _1)+a_1^6 \left( \eta _1 \left( \epsilon _1^6+114 \epsilon _0 \epsilon _2 \epsilon _1^4+720 \epsilon _0^2 \epsilon _2^2 \epsilon _1^2+272 \epsilon _0^3 \epsilon _2^3\right) +42 \eta _2 \epsilon _0 \epsilon _1 \left( 3 \epsilon _1^4+56 \epsilon _0 \epsilon _2 \epsilon _1^2+88 \epsilon _0^2 \epsilon _2^2\right) \right) \\ & \quad \quad +a_1^4 (720 \eta _2^2 \epsilon _1 \epsilon _0^3+30 \eta _2 \epsilon _0 \left( \left( 15 \eta _0+1\right) \epsilon _1 \left( \epsilon _1^2+4 \epsilon _0 \epsilon _2\right) +\eta _1 \epsilon _0 \left( 37 \epsilon _1^2+32 \epsilon _0 \epsilon _2\right) \right) \\ & \quad \quad +\eta _1 \left( \left( 15 \eta _0+1\right) \left( \epsilon _1^4+22 \epsilon _0 \epsilon _2 \epsilon _1^2+16 \epsilon _0^2 \epsilon _2^2\right) +15 \eta _1 \epsilon _0 \epsilon _1 \left( 7 \epsilon _1^2+32 \epsilon _0 \epsilon _2\right) \right) ) \\ & \quad \quad +3 a_1^2 (\eta _1 (30 \eta _1^2 \epsilon _0^2+6 \left( 15 \eta _0+1\right) \eta _1 \epsilon _0 \epsilon _1+\eta _0 \left( 15 \eta _0+2\right) \left( \epsilon _1^2+2 \epsilon _0 \epsilon _2\right) ) \\ & \quad \quad +6 \eta _2 \epsilon _0 \left( \eta _0 \left( 15 \eta _0+2\right) \epsilon _1+2 \left( 15 \eta _0+1\right) \eta _1 \epsilon _0\right) )=0, \end{aligned}$$

$$\begin{aligned} & \displaystyle \underline{\Theta ^{2} coeff:}\\ & \quad \quad \left( 3 \eta _1 \epsilon _1 \epsilon _2+4 \eta _2 \left( \epsilon _1^2+2 \epsilon _0 \epsilon _2\right) \right) \left( \alpha _1 a_2^2+a_3^2 \alpha _2+a_3 a_2 \beta _2\right) \\ & \quad \quad +a_1 \left( a_2 \beta _1+a_3 \beta _3+a_4\right) \left( 3 \eta _1 \epsilon _1 \epsilon _2+4 \eta _2 \left( \epsilon _1^2+2 \epsilon _0 \epsilon _2\right) \right) +a_1^6 \,(21 \eta _1 \epsilon _1 \epsilon _2 \left( 3 \epsilon _1^4+56 \epsilon _0 \epsilon _2 \epsilon _1^2+88 \epsilon _0^2 \epsilon _2^2\right) \\ & \quad \quad +8 \eta _2 \left( 8 \epsilon _1^6+387 \epsilon _0 \epsilon _2 \epsilon _1^4+1665 \epsilon _0^2 \epsilon _2^2 \epsilon _1^2+496 \epsilon _0^3 \epsilon _2^3\right) ) \\ & \quad \quad +a_1^4 (30 \eta _2^2 \epsilon _0^2 \left( 73 \epsilon _1^2+56 \epsilon _0 \epsilon _2\right) +\eta _2 (8 \left( 15 \eta _0+1\right) \left( 2 \epsilon _1^4+29 \epsilon _0 \epsilon _2 \epsilon _1^2+17 \epsilon _0^2 \epsilon _2^2\right) \\ & \quad \quad +15 \eta _1 \epsilon _0 \epsilon _1 \left( 103 \epsilon _1^2+368 \epsilon _0 \epsilon _2\right) ) +15 \eta _1 \left( \left( 15 \eta _0+1\right) \epsilon _1 \epsilon _2 \left( \epsilon _1^2+4 \epsilon _0 \epsilon _2\right) +4 \eta _1 \left( \epsilon _1^4+16 \epsilon _0 \epsilon _2 \epsilon _1^2+10 \epsilon _0^2 \epsilon _2^2\right) \right) ) \\ & \quad \quad +3 a_1^2 (12 \left( 15 \eta _0+1\right) \eta _2^2 \epsilon _0^2 +2 \eta _2 \left( 90 \eta _1^2 \epsilon _0^2+15 \left( 15 \eta _0+1\right) \eta _1 \epsilon _0 \epsilon _1+2 \eta _0 \left( 15 \eta _0+2\right) \left( \epsilon _1^2+2 \epsilon _0 \epsilon _2\right) \right) \\ & \quad \quad +\eta _1 \left( 75 \eta _1^2 \epsilon _0 \epsilon _1+4 \left( 15 \eta _0+1\right) \eta _1 \left( \epsilon _1^2+2 \epsilon _0 \epsilon _2\right) +3 \eta _0 \left( 15 \eta _0+2\right) \epsilon _1 \epsilon _2\right) )=0, \\ & \displaystyle \underline{\Theta ^{3} coeff:}\\ & \quad \quad 2 \epsilon _2 \left( \eta _1 \epsilon _2+5 \eta _2 \epsilon _1\right) \left( \alpha _1 a_2^2+a_3^2 \alpha _2+a_3 a_2 \beta _2\right) +2 a_1 \epsilon _2 \left( a_2 \beta _1+a_3 \beta _3+a_4\right) \left( \eta _1 \epsilon _2+5 \eta _2 \epsilon _1\right) \\ & \quad \quad +14 a_1^6 \epsilon _2 \left( \eta _1 \epsilon _2 \left( 43 \epsilon _1^4+256 \epsilon _0 \epsilon _2 \epsilon _1^2+88 \epsilon _0^2 \epsilon _2^2\right) +5 \eta _2 \epsilon _1 \left( 19 \epsilon _1^4+256 \epsilon _0 \epsilon _2 \epsilon _1^2+328 \epsilon _0^2 \epsilon _2^2\right) \right) \\ & \quad \quad +5 a_1^4 (6 \eta _2^2 \epsilon _0 \epsilon _1 \left( 83 \epsilon _1^2+276 \epsilon _0 \epsilon _2\right) +\eta _2 (2 \left( 15 \eta _0+1\right) \epsilon _1 \epsilon _2 \left( 13 \epsilon _1^2+44 \epsilon _0 \epsilon _2\right) \\ & \quad \quad +3 \eta _1 \left( 45 \epsilon _1^4+614 \epsilon _0 \epsilon _2 \epsilon _1^2+344 \epsilon _0^2 \epsilon _2^2\right) ) +\eta _1 \epsilon _2 \left( 2 \left( 15 \eta _0+1\right) \epsilon _2 \left( 5 \epsilon _1^2+4 \epsilon _0 \epsilon _2\right) +3 \eta _1 \epsilon _1 \left( 37 \epsilon _1^2+132 \epsilon _0 \epsilon _2\right) \right) ) \\ & \quad \quad +3 a_1^2 (4 \eta _2^2 \epsilon _0 \left( 7 \left( 15 \eta _0+1\right) \epsilon _1+75 \eta _1 \epsilon _0\right) +2 \eta _2 (210 \eta _1^2 \epsilon _0 \epsilon _1+9 \left( 15 \eta _0+1\right) \eta _1 \left( \epsilon _1^2+2 \epsilon _0 \epsilon _2\right) \\ & \quad \quad +5 \eta _0 \left( 15 \eta _0+2\right) \epsilon _1 \epsilon _2)+\eta _1 \left( 2 \eta _0 \left( 15 \eta _0+2\right) \epsilon _2^2+10 \left( 15 \eta _0+1\right) \eta _1 \epsilon _1 \epsilon _2+45 \eta _1^2 \left( \epsilon _1^2+2 \epsilon _0 \epsilon _2\right) \right) )=0, \end{aligned}$$

31 $$\begin{aligned} \begin{aligned}&\displaystyle \underline{\Theta ^{4}coeff:}\\&\quad \quad 3 (2 \eta _2 \epsilon _2^2 \left( \alpha _1 a_2^2+a_3^2 \alpha _2+a_3 a_2 \beta _2\right) +2 a_1 \eta _2 \epsilon _2^2 \left( a_2 \beta _1+a_3 \beta _3+a_4\right) +14 a_1^6 \epsilon _2^2 (50 \eta _1 \epsilon _1 \epsilon _2 \left( \epsilon _1^2+2 \epsilon _0 \epsilon _2\right) \\&\quad \quad +\eta _2 \left( 193 \epsilon _1^4+956 \epsilon _0 \epsilon _2 \epsilon _1^2+288 \epsilon _0^2 \epsilon _2^2\right) )+5 a_1^4 (\eta _1 \epsilon _2^2 \left( 4 \left( 15 \eta _0+1\right) \epsilon _1 \epsilon _2+\eta _1 \left( 101 \epsilon _1^2+76 \epsilon _0 \epsilon _2\right) \right) \\&\quad \quad +\eta _2 \epsilon _2 \left( 2 \left( 15 \eta _0+1\right) \epsilon _2 \left( 11 \epsilon _1^2+8 \epsilon _0 \epsilon _2\right) +\eta _1 \epsilon _1 \left( 313 \epsilon _1^2+1028 \epsilon _0 \epsilon _2\right) \right) +64 \eta _2^2 \left( \epsilon _1^4+13 \epsilon _0 \epsilon _2 \epsilon _1^2+7 \epsilon _0^2 \epsilon _2^2\right) ) \\&\quad \quad +a_1^2 (150 \eta _2^3 \epsilon _0^2+\eta _2^2 \left( 16 \left( 15 \eta _0+1\right) \left( \epsilon _1^2+2 \epsilon _0 \epsilon _2\right) +675 \eta _1 \epsilon _0 \epsilon _1\right) +6 \eta _2 (\eta _0 \left( 15 \eta _0+2\right) \epsilon _2^2 \\&\quad \quad +7 \left( 15 \eta _0+1\right) \eta _1 \epsilon _1 \epsilon _2+40 \eta _1^2 \left( \epsilon _1^2+2 \epsilon _0 \epsilon _2\right) )+3 \eta _1^2 \epsilon _2 \left( \left( 30 \eta _0+2\right) \epsilon _2+35 \eta _1 \epsilon _1\right) ))=0,\\&\displaystyle \underline{\Theta ^{5} coeff:}\\&\quad \quad 3 a_1^2 (560 a_1^4 \epsilon _2^3 \left( \eta _1 \epsilon _2 \left( 2 \epsilon _1^2+\epsilon _0 \epsilon _2\right) +\eta _2 \epsilon _1 \left( 13 \epsilon _1^2+23 \epsilon _0 \epsilon _2\right) \right) +2 a_1^2 \epsilon _2 (2 \eta _1 \epsilon _2^2 \left( \left( 30 \eta _0+2\right) \epsilon _2+135 \eta _1 \epsilon _1\right) \\&\quad \quad +4 \eta _2 \epsilon _2 \left( 14 \left( 15 \eta _0+1\right) \epsilon _1 \epsilon _2+65 \eta _1 \left( 7 \epsilon _1^2+5 \epsilon _0 \epsilon _2\right) \right) +5 \eta _2^2 \epsilon _1 \left( 197 \epsilon _1^2+628 \epsilon _0 \epsilon _2\right) )+330 \eta _2^3 \epsilon _0 \epsilon _1 \\&\quad \quad +60 \eta _1^3 \epsilon _2^2 +3 \eta _2^2 \left( 12 \left( 15 \eta _0+1\right) \epsilon _1 \epsilon _2+125 \eta _1 \left( \epsilon _1^2+2 \epsilon _0 \epsilon _2\right) \right) +12 \eta _1 \eta _2 \epsilon _2 \left( \left( 30 \eta _0+2\right) \epsilon _2+45 \eta _1 \epsilon _1\right) )=0,\\&\displaystyle \underline{\Theta ^{6} coeff:} \\&\quad \quad 15 (56 a_1^6 \epsilon _2^4 \left( 3 \eta _1 \epsilon _1 \epsilon _2+\eta _2 \left( 35 \epsilon _1^2+16 \epsilon _0 \epsilon _2\right) \right) +2 a_1^4 \epsilon _2^2 (4 \left( 5 \eta _1^2+15 \eta _0 \eta _2+\eta _2\right) \epsilon _2^2 \\&\quad \quad +2 \eta _2 \epsilon _2 \left( 175 \eta _1 \epsilon _1+148 \eta _2 \epsilon _0\right) +423 \eta _2^2 \epsilon _1^2)+a_1^2 \eta _2 (60 \eta _1^2 \epsilon _2^2+36 \eta _2^2 \left( \epsilon _1^2+2 \epsilon _0 \epsilon _2\right) \\&\quad \quad +\eta _2 \epsilon _2 \left( \left( 60 \eta _0+4\right) \epsilon _2+165 \eta _1 \epsilon _1\right) ))=0,\\&\displaystyle \underline{\Theta ^{7} coeff:} \\&\quad \quad 90 \left( 8 a_1^6 \epsilon _2^5 \left( \eta _1 \epsilon _2+27 \eta _2 \epsilon _1\right) +8 a_1^4 \eta _2 \epsilon _2^3 \left( 5 \eta _1 \epsilon _2+16 \eta _2 \epsilon _1\right) +a_1^2 \eta _2^2 \epsilon _2 \left( 15 \eta _1 \epsilon _2+13 \eta _2 \epsilon _1\right) \right) =0,\\&\displaystyle \underline{\Theta ^{8} coeff:}\\&\quad \quad 630 a_1^2 \eta _2 \epsilon _2^2 \left( 2 a_1^2 \epsilon _2^2+\eta _2\right) \left( 4 a_1^2 \epsilon _2^2+\eta _2\right) =0. \end{aligned} \end{aligned}$$

### Derived analytic solutions

6. The system in Eq.(31) was solved using Mathematica 14 software and the following results were obtained:

#### Result (1):

32 $$\begin{aligned} \begin{aligned}&\displaystyle \eta _0= \frac{1}{15} \left( -5\, a_1^2 \left( \epsilon _1^2+8\, \epsilon _0 \,\epsilon _2\right) -1\right) ,\\&\displaystyle \eta _1= -4\, a_1^2\, \epsilon _1\, \epsilon _2, \\&\displaystyle \eta _2= -4\, a_1^2 \,\epsilon _2^2,\\&\displaystyle a_4= -\frac{\alpha _1 \,a_2^2+a_3^2\, \alpha _2+a_3\, a_2\, \beta _2}{a_1}-a_2 \,\beta _1-a_3\, \beta _3+a_1^5 \,\left( -\left( \epsilon _1^2-4\, \epsilon _0\, \epsilon _2\right) {}^2\right) +\frac{a_1}{5}. \end{aligned} \end{aligned}$$

7. Inserting the obtained parameters in Eq.(30) then into Eq.(27). Then, using Eqs.(7)–(25), the derived exact solutions for the (3+1)-dimensional Lax integrable KP-SKRE in Eq.(1), are:

I Bright soliton, singular soliton, combo bright-dark soliton, and hyperbolic solutions for $K=\epsilon _1^2-4\,\epsilon _0\,\epsilon _2>0$, $\epsilon _0\,\epsilon _2\ne 0$,  or  $\epsilon _1\,\epsilon _2 \ne 0$, $b_1\ne 0$, and $b_2\ne 0$:

33 $$\begin{aligned} \displaystyle Q_{1.1,1}(t,\,x,\,y,\,z)= & \displaystyle -\frac{1}{15}\left( 1+5a_1^2\,K\right) + a_1^2\,K \, \textrm{sech} ^2\left( \frac{1}{2} \sqrt{K} \left( a_4 t+a_1 x+a_2 y+a_3 z+\varepsilon \right) \right) , \end{aligned}$$

34 $$\begin{aligned} \displaystyle Q_{1.1,2}(t,\,x,\,y,\,z)= & \displaystyle -\frac{1}{15}\left( 1+5a_1^2\,K\right) - a_1^2\,K \textrm{csch} ^2\left( \frac{1}{2} \sqrt{K} \left( a_4 t+a_1 x+a_2 y+a_3 z+\varepsilon \right) \right) , \end{aligned}$$

35 $$\begin{aligned} \displaystyle Q_{1.1,3}^{\pm }(t,\,x,\,y,\,z)= & \displaystyle -\frac{1}{15}\left( 1+5a_1^2\,K\right) +\displaystyle 2\, a_1^2\,K \textrm{sech}^2\left( \sqrt{K} \left( a_4 t+a_1 x+a_2 y+a_3 z+\varepsilon \right) \right) \nonumber \\ & \mp \,\textrm{i}\,2\, a_1^2\,K\, \textrm{sech}\left( \sqrt{K} \left( a_4 t+a_1 x+a_2 y+a_3 z+\varepsilon \right) \right) \,\nonumber \\ & \textrm{tanh} \left( \sqrt{K} \left( a_4 t+a_1 x+a_2 y+a_3 z+\varepsilon \right) \right) , \end{aligned}$$

36 $$\begin{aligned} \displaystyle Q_{1.1,4}(t,\,x,\,y,\,z)= & \displaystyle -\frac{1}{15} +\frac{\,a_1^2 K}{6} \displaystyle -\frac{a_1^2 K}{4} \textrm{tanh}^2 \left( \frac{1}{4} \sqrt{K} \left( a_4 t+a_1 x+a_2 y+a_3 z+\varepsilon \right) \right) \nonumber \\ & \displaystyle -\frac{a_1^2 K}{4} \textrm{coth}^2 \left( \frac{1}{4} \sqrt{K} \left( a_4 t+a_1 x+a_2 y+a_3 z+\varepsilon \right) \right) , \end{aligned}$$

37 $$\begin{aligned} \displaystyle Q_{1.1,5}^{\pm }(t,\,x,\,y,\,z)= & \displaystyle -\frac{1}{15}\nonumber \\ & +\displaystyle \frac{a_1^2 K}{6\,\left( b_1 \textrm{sinh} \left( \sqrt{K} \left( a_4 t+a_1 x+a_2 y+a_3 z+\varepsilon \right) \right) +b_2\right) {}^2} \left( -2 b_2^2-11\,b_1^2\nonumber \right. \\ & -b_1^2 \textrm{cosh} \left( 2 \sqrt{K} \left( a_4 t+a_1 x+a_2 y+a_3 z+\varepsilon \right) \right) \nonumber \\ & +8 b_1 b_2 \textrm{sinh} \left( \sqrt{K} \left( a_4 t+a_1 x+a_2 y+a_3 z+\varepsilon \right) \right) \nonumber \\ & \pm \,\left. 12\,b_1 \sqrt{b_1^2+b_2^2} \textrm{cosh} \left( \sqrt{K} \left( a_4 t+a_1 x+a_2 y+a_3 z+\varepsilon \right) \right) \right) , \end{aligned}$$

38 $$\begin{aligned} \displaystyle Q_{1.1,6}^{+}(t,\,x,\,y,\,z)= & \displaystyle -\frac{8 a_1^2 \epsilon _0 \epsilon _1 \epsilon _2}{\sqrt{K} \textrm{coth} \left( \frac{1}{2} \sqrt{K} \left( a_4 t+a_1 x+a_2 y+a_3 z+\varepsilon \right) \right) -\epsilon _1}\nonumber \\ & \displaystyle -\frac{16 a_1^2 \epsilon _0^2 \epsilon _2^2}{\left( \sqrt{K} \textrm{coth} \left( \frac{1}{2} \sqrt{K} \left( a_4 t+a_1 x+a_2 y+a_3 z+\varepsilon \right) \right) -\epsilon _1\right) {}^2}-\frac{1}{15} \left( 5 a_1^2 \left( \epsilon _1^2+8 \epsilon _0 \epsilon _2\right) +1\right) . \end{aligned}$$

39 $$\begin{aligned} \displaystyle Q_{1.1,6}^{-}(t,\,x,\,y,\,z)= & \displaystyle -\frac{8 a_1^2 \epsilon _0 \epsilon _1 \epsilon _2}{\sqrt{K} \textrm{tanh} \left( \frac{1}{2} \sqrt{K} \left( a_4 t+a_1 x+a_2 y+a_3 z+\varepsilon \right) \right) -\epsilon _1}\nonumber \\ & \displaystyle -\frac{16 a_1^2 \epsilon _0^2 \epsilon _2^2}{\left( \sqrt{K} \textrm{tanh} \left( \frac{1}{2} \sqrt{K} \left( a_4 t+a_1 x+a_2 y+a_3 z+\varepsilon \right) \right) -\epsilon _1\right) {}^2}-\frac{1}{15} \left( 5 a_1^2 \left( \epsilon _1^2+8 \epsilon _0 \epsilon _2\right) +1\right) . \end{aligned}$$

II Singular periodic solutions for $K=\epsilon _1^2-4\,\epsilon _0\,\epsilon _2<0$, $\epsilon _0\,\epsilon _2\ne 0$,  or  $\epsilon _1\,\epsilon _2 \ne 0$, and $b_1^2-b_2^2>0$:

40 $$\begin{aligned} \displaystyle Q_{1.2,1}(t,\,x,\,y,\,z)= & \displaystyle -\frac{1}{15}\left( 1+5 \, a_1^2 \,K \right) +a_1^2 K \textrm{sec} ^2\left( \frac{1}{2} \sqrt{-K} \left( a_4 t+a_1 x+a_2 y+a_3 z+\varepsilon \right) \right) , \end{aligned}$$

41 $$\begin{aligned} \displaystyle Q_{1.2,2}(t,\,x,\,y,\,z)= & \displaystyle -\frac{1}{15}\left( 1+5 \, a_1^2 \,K \right) +a_1^2 K \textrm{csc} ^2\left( \frac{1}{2} \sqrt{-K} \left( a_4 t+a_1 x+a_2 y+a_3 z+\varepsilon \right) \right) , \end{aligned}$$

42 $$\begin{aligned} \displaystyle Q_{1.2,3}^{\pm }(t,\,x,\,y,\,z)= & \displaystyle -\frac{1}{15}\left( 1+5 \, a_1^2 \,K \right) + \displaystyle 2\, a_1^2 \,K \, \textrm{sec}^2 \left( \sqrt{-K} \left( a_4 t+a_1 x+a_2 y+a_3 z+\varepsilon \right) \right) \nonumber \\ & \pm \displaystyle 2\, a_1^2 \,K \textrm{tan} \left( \sqrt{-K} \left( a_4 t+a_1 x+a_2 y+a_3 z+\varepsilon \right) \right) \, \nonumber \\ & \textrm{sec} \left( \sqrt{-K} \left( a_4 t+a_1 x+a_2 y+a_3 z+\varepsilon \right) \right) , \end{aligned}$$

43 $$\begin{aligned} \displaystyle Q_{1.2,4}(t,\,x,\,y,\,z)= & \displaystyle -\frac{1}{15}+\frac{ a_1^2 K}{6} \displaystyle + \frac{a_1^2 K}{4} \textrm{cot}^2 \left( \frac{1}{4} \sqrt{-K} \left( a_4 t+a_1 x+a_2 y+a_3 z+\varepsilon \right) \right) \nonumber \\ & \displaystyle + \frac{a_1^2 K}{4} \textrm{tan}^2 \left( \frac{1}{4} \sqrt{-K} \left( a_4 t+a_1 x+a_2 y+a_3 z+\varepsilon \right) \right) , \end{aligned}$$

44 $$\begin{aligned} \displaystyle Q_{1.2,5}^{\pm }(t,\,x,\,y,\,z)= & \displaystyle -\frac{1}{15}\nonumber \\ & +\displaystyle \frac{a_1^2 K}{6\,\left( b_1 \textrm{sin} \left( \sqrt{-K} \left( a_4 t+a_1 x+a_2 y+a_3 z+\varepsilon \right) \right) +b_2\right) {}^2} \left( -2 b_2^2+11\,b_1^2 \nonumber \right. \\ & +b_1^2 \textrm{cos} \left( 2 \sqrt{-K} \left( a_4 t+a_1 x+a_2 y+a_3 z+\varepsilon \right) \right) \nonumber \\ & +8 b_1 b_2 \textrm{sin} \left( \sqrt{-K} \left( a_4 t+a_1 x+a_2 y+a_3 z+\varepsilon \right) \right) \nonumber \\ & \mp \left. \,12\,b_1 \sqrt{b_1^2-b_2^2} \textrm{cos} \left( \sqrt{-K} \left( a_4 t+a_1 x+a_2 y+a_3 z+\varepsilon \right) \right) \right) , \end{aligned}$$

45 $$\begin{aligned} & \displaystyle Q_{1.2,6}^{\pm }(t,\,x,\,y,\,z)\nonumber \\ & \quad =\displaystyle -\frac{1}{15}-\frac{a_1^2}{3}\,\left( \epsilon _1^2+8 \epsilon _0 \epsilon _2\right) \nonumber \\ & \quad \quad + \displaystyle \frac{8 a_1^2\epsilon _0 \epsilon _1 \epsilon _2 \textrm{cos} \left( \sqrt{-K} \left( a_4 t+a_1 x+a_2 y+a_3 z+\varepsilon \right) \right) }{\sqrt{-K} \left( \textrm{sin} \left( \sqrt{-K} \left( a_4 t+a_1 x+a_2 y+a_3 z+\varepsilon \right) \right) \pm 1\right) +\epsilon _1 \textrm{cos} \left( \sqrt{-K} \left( a_4 t+a_1 x+a_2 y+a_3 z+\varepsilon \right) \right) }\nonumber \\ & \quad \quad \displaystyle -\frac{16a_1^2 \epsilon _0 \epsilon _2 \textrm{cos} ^2\left( \sqrt{-K} \left( a_4 t+a_1 x+a_2 y+a_3 z+\varepsilon \right) \right) }{\left( \sqrt{-K} \left( \textrm{sin} \left( \sqrt{-K} \left( a_4 t+a_1 x+a_2 y+a_3 z+\varepsilon \right) \right) \pm 1\right) +\epsilon _1 \textrm{cos} \left( \sqrt{-K} \left( a_4 t+a_1 x+a_2 y+a_3 z+\varepsilon \right) \right) \right) {}^2}, \end{aligned}$$

46 $$\begin{aligned} & \displaystyle Q_{1.2,7}^{\pm }(t,\,x,\,y,\,z)\nonumber \\ & \quad = \displaystyle -\frac{1}{15}-\frac{a_1^2}{3}\left( \epsilon _1^2+8 \epsilon _0 \epsilon _2\right) \nonumber \\ & \quad \quad \displaystyle +\frac{8a_1^2 \epsilon _0 \epsilon _1 \epsilon _2 \textrm{sin} \left( \sqrt{-K} \left( a_4 t+a_1 x+a_2 y+a_3 z+\varepsilon \right) \right) }{\epsilon _1 \textrm{sin} \left( \sqrt{-K} \left( a_4 t+a_1 x+a_2 y+a_3 z+\varepsilon \right) \right) -\sqrt{-K} \left( \textrm{cos} \left( \sqrt{-K} \left( a_4 t+a_1 x+a_2 y+a_3 z+\varepsilon \right) \right) \mp 1\right) }\nonumber \\ & \quad \quad \displaystyle -\frac{16 a_1^2\epsilon _0 \epsilon _2 \textrm{sin} ^2\left( \sqrt{-K} \left( a_4 t+a_1 x+a_2 y+a_3 z+\varepsilon \right) \right) }{\left( \sqrt{-K} \left( \textrm{cos} \left( \sqrt{-K} \left( a_4 t+a_1 x+a_2 y+a_3 z+\varepsilon \right) \right) \mp 1\right) -\epsilon _1 \textrm{sin} \left( \sqrt{-K} \left( a_4 t+a_1 x+a_2 y+a_3 z+\varepsilon \right) \right) \right) {}^2}. \end{aligned}$$

III Exponential solutions with *v* as arbitrary constant:

For $$\epsilon _0=0,\,\epsilon _1\ne 0$$, and $$\epsilon _2\ne 0$$:

47 $$\begin{aligned} \displaystyle Q_{1.3,1}(t,\,x,\,y,\,z)= & \displaystyle -\frac{1}{15}\left( 1+5\,a_1^2 \epsilon _1^2\right) +\displaystyle \frac{4 a_1^2\, v \,\epsilon _1^2\, \displaystyle \textrm{e}^{\epsilon _1 \left( a_4 t+a_1 x+a_2 y+a_3 z+\varepsilon \right) }}{\left( v \, \displaystyle \textrm{e}^{\epsilon _1 \left( a_4 t+a_1 x+a_2 y+a_3 z+\varepsilon \right) }+1\right) {}^2}, \end{aligned}$$

48 $$\begin{aligned} \displaystyle Q_{1.3,2}(t,\,x,\,y,\,z)= & \displaystyle -\frac{1}{15}\left( 1+5\,a_1^2 \epsilon _1^2\right) + \frac{4 a_1^2\, v\, \epsilon _1^2\, \displaystyle \textrm{e}^{\epsilon _1 \left( a_4 t+a_1 x+a_2 y+a_3 z+\varepsilon \right) }}{\left( \displaystyle \textrm{e}^{\epsilon _1 \left( a_4 t+a_1 x+a_2 y+a_3 z+\varepsilon \right) }+v\right) {}^2}. \end{aligned}$$

IV Rational solutions:
  1. For $$\epsilon _0=\epsilon _{1}=0$$,  and $$\,\epsilon _{2}\ne 0$$:
    49 $$\begin{aligned} \displaystyle Q_{1.4,1}^{\pm }(t,\,x,\,y,\,z)= \displaystyle -\frac{4 a_1^2}{\left( a_4 t+a_1 x+a_2 y+a_3 z+\varepsilon \right) {}^2}-\frac{1}{15}. \end{aligned}$$
  2. For $$\epsilon _0\ne 0,\,\epsilon _{1}\ne 0$$,  and $$\,\epsilon _{2}=\displaystyle \frac{\epsilon _1^2}{4\,\epsilon _0}$$:
    50 $$\begin{aligned} \displaystyle Q_{1.4,2}(t,\,x,\,y,\,z)= \displaystyle -\frac{4 a_1^2}{\left( a_4 t+a_1 x+a_2 y+a_3 z+\varepsilon \right) {}^2}-\frac{1}{15}. \end{aligned}$$

#### Result (2):

51 $$\begin{aligned} \begin{aligned}&\displaystyle \eta _1= -2\, a_1^2\, \epsilon _1 \,\epsilon _2,\\&\eta _2= -2 \,a_1^2\, \epsilon _2^2,\\&a_4= -\left( a_3 \, \beta _3+a_2 \, \beta _1+3 \,a_1 \,\eta _0 \left( 15\, \eta _0+2\right) +a_1^3 \,\left( 15\, \eta _0+1\right) \left( \epsilon _1^2+8\, \epsilon _0\, \epsilon _2\right) +a_1^5 \left( \epsilon _1^4+22\, \epsilon _0\, \epsilon _2 \,\epsilon _1^2+76\, \epsilon _0^2 \,\epsilon _2^2\right) \right) \\&-\displaystyle \frac{a_2^2 \,\alpha _1+a_3^2\, \alpha _2+a_2 \,a_3 \,\beta _2}{a_1}. \end{aligned} \end{aligned}$$

8 Let $\eta _0=-\displaystyle \frac{1}{2} a_1^2 \epsilon _1^2$ then inserting the obtained parameters in Eq.(30) then into Eq.(27) and using Eqs.(7)–(25), the analytic solutions for the (3+1)-dimensional Lax integrable KP-SKRE in Eq.(1), are:

I Bright soliton, singular soliton, combo bright-dark soliton, and hyperbolic solutions for $K=\epsilon _1^2-4\,\epsilon _0\,\epsilon _2>0$, $\epsilon _0\,\epsilon _2\ne 0$,  or  $\epsilon _1\,\epsilon _2 \ne 0$, $b_1\ne 0$, and $b_2\ne 0$:

52 $$\begin{aligned} \displaystyle Q_{2.1,1}(t,\,x,\,y,\,z)= & \displaystyle \frac{1}{2} a_1^2 \,K \left( \textrm{sech} ^2\left( \frac{1}{2} \sqrt{K} \left( a_4 t+a_1 x+a_2 y+a_3 z+\varepsilon \right) \right) -1\right) , \end{aligned}$$

53 $$\begin{aligned} \displaystyle Q_{2.1,2}(t,\,x,\,y,\,z)= & - \displaystyle \frac{1}{2} a_1^2 \,K \textrm{coth} ^2\left( \frac{1}{2} \sqrt{K} \left( a_4 t+a_1 x+a_2 y+a_3 z+\varepsilon \right) \right) , \end{aligned}$$

54 $$\begin{aligned} \displaystyle Q_{2.1,3}^{\pm }(t,\,x,\,y,\,z)= & \displaystyle - \frac{1}{2} a_1^2 \,K\ +a_1^2 \,K\, \textrm{sech}^2\left( \sqrt{K} \left( a_4 t+a_1 x+a_2 y+a_3 z+\varepsilon \right) \right) \nonumber \\ & \mp \,\,\textrm{i}\,a_1^2 \,K\, \textrm{sech}\left( \sqrt{K} \left( a_4 t+a_1 x+a_2 y+a_3 z+\varepsilon \right) \right) \,\textrm{tanh} \left( \sqrt{K} \left( a_4 t+a_1 x+a_2 y+a_3 z+\varepsilon \right) \right) , \end{aligned}$$

55 $$\begin{aligned} \displaystyle Q_{2.1,4}(t,\,x,\,y,\,z)= & -\displaystyle \frac{1}{4} a_1^2 \,K -\displaystyle \frac{1}{8} a_1^2 \,K \textrm{tanh}^2 \left( \frac{1}{4} \sqrt{K} \left( a_4 t+a_1 x+a_2 y+a_3 z+\varepsilon \right) \right) \nonumber \\ & -\displaystyle \frac{1}{8} a_1^2 \,K \textrm{coth}^2 \left( \frac{1}{4} \sqrt{K} \left( a_4 t+a_1 x+a_2 y+a_3 z+\varepsilon \right) \right) , \end{aligned}$$

56 $$\begin{aligned} \displaystyle Q_{2.1,5}^{\pm }(t,\,x,\,y,\,z)= & \displaystyle -\frac{a_1^2 K \left( -b_1 \textrm{cosh} \left( \sqrt{K} \left( a_4 t+a_1 x+a_2 y+a_3 z+\varepsilon \right) \right) \pm \,\sqrt{b_1^2+b_2^2}\right) {}^2}{2 \left( b_1 \textrm{sinh} \left( \sqrt{K} \left( a_4 t+a_1 x+a_2 y+a_3 z+\varepsilon \right) \right) +b_2\right) {}^2}, \end{aligned}$$

57 $$\begin{aligned} \displaystyle Q_{2.1,6}^{+}(t,\,x,\,y,\,z)= & \displaystyle -\frac{a_1^2 K \left( \sqrt{K}-\epsilon _1 \textrm{coth} \left( \frac{1}{2} \sqrt{K} \left( a_4 t+a_1 x+a_2 y+a_3 z+\varepsilon \right) \right) \right) {}^2}{2 \left( \epsilon _1-\sqrt{K} \textrm{coth} \left( \frac{1}{2} \sqrt{K} \left( a_4 t+a_1 x+a_2 y+a_3 z+\varepsilon \right) \right) \right) {}^2}, \end{aligned}$$

58 $$\begin{aligned} \displaystyle Q_{2.1,6}^{-}(t,\,x,\,y,\,z)= & -\frac{a_1^2 K \left( \sqrt{K}-\epsilon _1 \textrm{tanh} \left( \frac{1}{2} \sqrt{K} \left( a_4 t+a_1 x+a_2 y+a_3 z+\varepsilon \right) \right) \right) {}^2}{2 \left( \epsilon _1-\sqrt{K} \textrm{tanh} \left( \frac{1}{2} \sqrt{K} \left( a_4 t+a_1 x+a_2 y+a_3 z+\varepsilon \right) \right) \right) {}^2}. \end{aligned}$$

II Singular periodic solutions for $K=\epsilon _1^2-4\,\epsilon _0\,\epsilon _2<0$, $\epsilon _0\,\epsilon _2\ne 0$,  or  $\epsilon _1\,\epsilon _2 \ne 0$, and $b_1^2-b_2^2>0$:

59 $$\begin{aligned} \displaystyle Q_{2.2,1}(t,\,x,\,y,\,z)= & \displaystyle \frac{1}{2} a_1^2 \,K \textrm{tan} ^2\left( \frac{1}{2} \sqrt{-K} \left( a_4 t+a_1 x+a_2 y+a_3 z+\varepsilon \right) \right) , \end{aligned}$$

60 $$\begin{aligned} \displaystyle Q_{2.2,2}(t,\,x,\,y,\,z)= & \displaystyle \frac{1}{2} a_1^2 \,K \textrm{cot} ^2\left( \frac{1}{2} \sqrt{-K} \left( a_4 t+a_1 x+a_2 y+a_3 z+\varepsilon \right) \right) , \end{aligned}$$

61 $$\begin{aligned} \displaystyle Q_{2.2,3}^{\pm }(t,\,x,\,y,\,z)= & - \displaystyle \frac{1}{2} a_1^2 K +a_1^2 \,K\, \textrm{sec}^2\left( \sqrt{-K} \left( a_4 t+a_1 x+a_2 y+a_3 z+\varepsilon \right) \right) \nonumber \\ & \pm \,\,a_1^2 \,K\, \textrm{sec}\left( \sqrt{-K} \left( a_4 t+a_1 x+a_2 y+a_3 z+\varepsilon \right) \right) \, \nonumber \\ & \textrm{tan} \left( \sqrt{-K} \left( a_4 t+a_1 x+a_2 y+a_3 z+\varepsilon \right) \right) , \end{aligned}$$

62 $$\begin{aligned} \displaystyle Q_{2.2,4}(t,\,x,\,y,\,z)= & -\displaystyle \frac{1}{4} a_1^2\,K +\displaystyle \frac{1}{8} a_1^2\,K \textrm{cot}^2 \left( \frac{1}{4} \sqrt{-K} \left( a_4 t+a_1 x+a_2 y+a_3 z+\varepsilon \right) \right) \nonumber \\ & +\displaystyle \frac{1}{8} a_1^2\,K \textrm{tan}^2 \left( \frac{1}{4} \sqrt{-K} \left( a_4 t+a_1 x+a_2 y+a_3 z+\varepsilon \right) \right) , \end{aligned}$$

63 $$\begin{aligned} \displaystyle Q_{2.2,5}^{\pm }(t,\,x,\,y,\,z)= & \frac{a_1^2 K \left( -\,b_1 \textrm{cos} \left( \sqrt{-K} \left( a_4 t+a_1 x+a_2 y+a_3 z+\varepsilon \right) \right) \pm \,\sqrt{b_1^2-b_2^2}\right) {}^2}{2 \left( b_1 \textrm{sin} \left( \sqrt{-K} \left( a_4 t+a_1 x+a_2 y+a_3 z+\varepsilon \right) \right) +b_2\right) {}^2}, \end{aligned}$$

64 $$\begin{aligned} \displaystyle Q_{2.2,6}^{+}(t,\,x,\,y,\,z)= & \displaystyle \frac{a_1^2 K \left( \sqrt{-K}-\epsilon _1 \textrm{tan} \left( \frac{1}{2} \sqrt{-K} \left( a_4 t+a_1 x+a_2 y+a_3 z+\varepsilon \right) \right) \right) {}^2}{2 \left( \epsilon _1+\sqrt{-K} \textrm{tan} \left( \frac{1}{2} \sqrt{-K} \left( a_4 t+a_1 x+a_2 y+a_3 z+\varepsilon \right) \right) \right) {}^2}. \end{aligned}$$

65 $$\begin{aligned} \displaystyle Q_{2.2,6}^{-}(t,\,x,\,y,\,z)= & \displaystyle \frac{a_1^2 K \left( \sqrt{-K}+\epsilon _1 \textrm{cot} \left( \frac{1}{2} \sqrt{-K} \left( a_4 t+a_1 x+a_2 y+a_3 z+\varepsilon \right) \right) \right) {}^2}{2 \left( \epsilon _1-\sqrt{-K} \textrm{cot} \left( \frac{1}{2} \sqrt{-K} \left( a_4 t+a_1 x+a_2 y+a_3 z+\varepsilon \right) \right) \right) {}^2} \end{aligned}$$

III Exponential solutions with *v* as arbitrary constant:

For $$\epsilon _0=0,\,\epsilon _1\ne 0$$, and $$\epsilon _2\ne 0$$:

66 $$\begin{aligned} \displaystyle Q_{2.3,1}(t,\,x,\,y,\,z)= & \displaystyle \,-\frac{a_1^2 \epsilon _1^2 \left( v \ \textrm{e}^{\epsilon _1 \left( a_4 t+a_1 x+a_2 y+a_3 z+\varepsilon \right) }-1\right) {}^2}{2 \left( v \textrm{e}^{\epsilon _1 \left( a_4 t+a_1 x+a_2 y+a_3 z+\varepsilon \right) }+1\right) {}^2}, \end{aligned}$$

67 $$\begin{aligned} \displaystyle Q_{2.3,2}(t,\,x,\,y,\,z)= & \displaystyle \,-\frac{a_1^2 \epsilon _1^2 \left( \ \textrm{e}^{\epsilon _1 \left( a_4 t+a_1 x+a_2 y+a_3 z+\varepsilon \right) }-v\right) {}^2}{2 \left( \textrm{e}^{\epsilon _1 \left( a_4 t+a_1 x+a_2 y+a_3 z+\varepsilon \right) }+v\right) {}^2}. \end{aligned}$$

IV Rational solutions:
  1. For $$\epsilon _0=\epsilon _{1}=0$$,  and $$\,\epsilon _{2}\ne 0$$:
    68 $$\begin{aligned} \displaystyle Q_{2.4,1}^{\pm }(t,\,x,\,y,\,z)= \displaystyle -\frac{2 a_1^2}{\left( a_4 t+a_1 x+a_2 y+a_3 z+\varepsilon \right) {}^2}. \end{aligned}$$
  2. For $$\epsilon _0\ne 0,\,\epsilon _{1}\ne 0$$,  and $$\,\epsilon _{2}=\frac{\epsilon _1^2}{4\,\epsilon _0}$$:
    69 $$\begin{aligned} \displaystyle Q_{2.4,2}(t,\,x,\,y,\,z)=- \,\displaystyle \, \frac{2\, a_1^2}{\left( a_4 t+a_1 x+a_2 y+a_3 z+\varepsilon \right) {}^2}. \end{aligned}$$

Many other forms of exact solutions can be obtained using the Bäcklund transformation of IGREMM in Eq.(26). The visualization of selected derived solutions is in sect. 5.

## Modulation instability study

The standard linear stability analysis is applied to study the modulation instability (MI) of Eq.(1) to get the restrictions under which MI happens and its impact on the wave forms’ dynamics. This analysis formulates a solution at steady-state (SS) to Eq.(1) and then introduces perturbations to the solution at SS and concludes the gain spectrum (GS) of MI^40–43^.

1. Assuming the solution of Eq. (1) at SS is as follows:
  70 $$\begin{aligned} Q= \sqrt{{\mathcal {M}}} , \end{aligned}$$
  where ${\mathcal {M}}$ is a constant with real value representing the normalized power. The SS solution in Eq.(70) satisfies Eq.(1).
2. By introducing a small perturbation in Eq.(70), the perturbed SS solution can be written as:
  71 $$\begin{aligned} Q(t,x,y,z)=\left[ R(t,x,y,z)+ \sqrt{{\mathcal {M}}}\right] , \end{aligned}$$
  with *R*(*t*, *x*, *y*, *z*) as the perturbation amplitude and $\mid R(t,x,y,z)\mid<<\sqrt{{\mathcal {M}}}$. The following equation is obtained by substituting Eq.(71) in Eq.(1) followed by a linearization w.r.t *R*(*t*, *x*, *y*, *z*) :
  72 $$\begin{aligned} \begin{aligned}&\displaystyle \left( 15 \sqrt{{\mathcal {M}}}+1\right) \frac{\partial ^4R(t,x,y,z)}{\partial x^4}+\left( 45 {\mathcal {M}}+6 \sqrt{\mathcal {M}}\right) \frac{\partial ^2R(t,x,y,z)}{\partial x^2}+\frac{\partial ^6R(t,x,y,z)}{\partial x^6} \\&\quad \quad +\displaystyle \alpha _1 \frac{\partial ^2R(t,x,y,z)}{\partial y^2}+\displaystyle \alpha _2 \frac{\partial ^2R(t,x,y,z)}{\partial z^2}+\beta _1 \frac{\partial ^2 R(t,x,y,z)}{\partial y\,\partial x}+\beta _2 \,\frac{\partial ^2 R(t,x,y,z)}{\partial z\,\partial y}\\&\quad \quad +\displaystyle \beta _3\,\frac{\partial ^2 R(t,x,y,z)}{\partial z\,\partial x}+\frac{\partial ^2 R(t,x,y,z)}{\partial t\,\partial x}=0. \end{aligned} \end{aligned}$$
3. Now, assume the solution of Eq.(72) as:
  73 $$\begin{aligned} \displaystyle R(t,x,y,z)=r_1 \, \textrm{e}^{-\textrm{i} (\omega \ t -L_1\ x-L_2\ y-L_3\ z)}+r_2 \, \textrm{e}^{\textrm{i} (\omega \ t-L_1\ x-L_2\ y-L_3\ z)}, \end{aligned}$$
  with $\omega$ as the frequency of perturbation, $r_1,r_2$ are constants, and $L_1, L_2,$ and $L_3$ are the perturbation wave numbers in $x,\,y,$ and $\,z$, respectively. Inserting Eq.(73) in Eq.(72) and then finding the coefficients of $\displaystyle \textrm{e}^{-\textrm{i} (\omega \ t -L_1\ x-L_2\ y-L_3\ z)}$ and $\displaystyle \textrm{e}^{\textrm{i} (\omega \ t-L_1\ x-L_2\ y-L_3\ z)}$ gives:
  74 $$\begin{aligned} \begin{aligned}&\displaystyle r_1 U_1+ r_2 U_2=0,\\&\displaystyle r_1 U_3+r_2 U_4=0, \end{aligned} \end{aligned}$$
  hence, the two equations are put in matrix form as follows:
  75 $$\begin{aligned} \begin{bmatrix} U_1 & U_2 \\ U_3 & U_4 \\ \end{bmatrix} \begin{bmatrix} r_1 \\ r_2 \\ \end{bmatrix}= \begin{bmatrix} 0 \\ 0 \\ \end{bmatrix}. \end{aligned}$$
  where,
  $$\begin{aligned} \begin{aligned} U_1&=U_4\\&=-\displaystyle \left( 15 \sqrt{{\mathcal {M}}}+1\right) L_1^4+\left( 45{\mathcal {M}}+6 \sqrt{{\mathcal {M}}}\right) L_1^2+\alpha _1 L_2^2+\alpha _2 L_3^2+L_1 \left( \beta _1 L_2+\beta _3 L_3-\omega \right) \displaystyle +\beta _2 L_2 L_3+L_1^6, \\&and,\\ U_2&=U_3=0.\\ \end{aligned} \end{aligned}$$
4. The determinant of the coefficient matrix in Eq.(75) must vanish to have a non-trivial solution for the system of equations and hence the dispersion relation is (for $L_1\ne 0$):
  76 $$\begin{aligned} \begin{aligned}&\omega ({\mathcal {M}}, {L_1})\\&\quad =\displaystyle -\left( 15 \sqrt{{\mathcal {M}}}+1\right) L_1^3+\left( 45 {\mathcal {M}}+6 \sqrt{{\mathcal {M}}}\right) L_1+\frac{\alpha _1 L_2^2+\alpha _2 L_3^2+\beta _2 L_3 L_2}{L_1}+\beta _1 L_2+\beta _3 L_3+L_1^5. \end{aligned} \end{aligned}$$

The perturbed solution in Eq.(71) is stable if $\omega$ in Eq.(76) has a real value. The real value of $\omega$ force the decaying of perturbations initiated at the SS. While the complex value of $\omega$ forces an exponential growth of any small perturbations initiated at the SS and hence become unstable, which is named MI. In MI, any deviation from SS amplifies and causes divergence^5,40,41,43^. The result of MI analysis in Eq.(76) in which $\omega$ is real valued, the solution at SS is always stable. Hence, the GS of MI $G({\mathcal {M}},$ $L_1)=2\,\text {Im}(\omega )$, measuring instability level, is zero because $\omega$ has zero imaginary part. So, perturbations initiated at the SS will vanish and the solution will be stable. The graphical illustration of the frequency of perturbation in Eq.(76) is shown in next section.

## Results and discussions

The application of IGREMM to the $(3+1)$-dimensional Lax integrable KP-SKRE in Eq.(1) generated abundant types of solutions. The derived solutions include bright, singular, and combo bright-dark soliton solutions along with exponential, singular periodic, hyperbolic, and rational solutions. In subsect. 5.1, the dynamics of some of the derived solutions are graphically illustrated, and the descriptions of their different characteristics, while in subsect. 5.2, the visualization of obtained results of MI analysis are presented.

According to our knowledge, the solutions previously obtained in the literature include multiple-soliton, and lump solutions in^26^. An exponential rational function solution in^35^. Single and combined Jacobi elliptic function solutions in^36^. Hybrid solutions in^37^. While the current work derived abundant novel solutions such as combo bright-dark soliton, singular soliton in addition to bright soliton, singular periodic, hyperbolic, exponential, and rational solutions for Eq.(1) using the improved generalized Riccati equation mapping method. The Bäcklund transformation of IGREMM in Eq.(26) can be used to construct additional novel forms of solution.

### Graphical illustrations and characteristics’ descriptions of some derived solutions

**Fig. 1:**
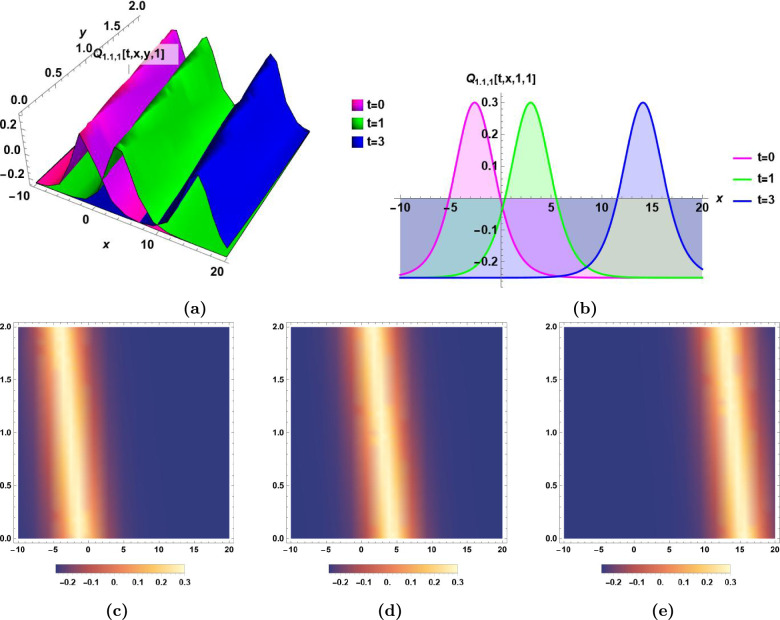
Bright soliton $Q_{1.1,1}(t,\,x,\,y,\,z)$ in Eq.(33) for $\epsilon _0=0.5,\,\epsilon _1=-1, \epsilon _2=-0.6,\,\alpha _1=0.9,\, \alpha _2=0.8,\,\beta _1=0.5,\,\beta _2=0.6,\,\beta _3=0.7,\,\varepsilon =0,\,a_1= 0.5,\,a_2=0.7,$ and $\,a_3=0.6$, **(a)** The dynamics of bright soliton $Q_{1.1,1}(t,\,x,\,y,\,1)$ with time *t* in 3D, **(b)** The dynamics of bright soliton $Q_{1.1,1}(t,\,x,\,1,\,1)$ with time *t* in 2D, **(c), (d),** and** (e)** The density plots of $Q_{1.1,1}(0,\,x,\,y,\,1)$, $Q_{1.1,1}(1,\,x,y,\,1)$, and $Q_{1.1,1}(3,\,x,\,y,\,1)$, respectively.

**Fig. 2:**
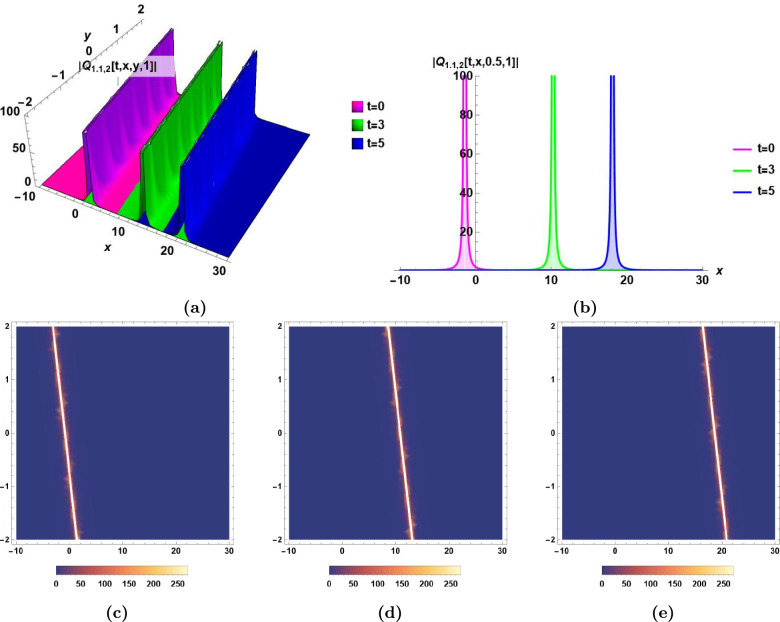
Singular soliton $|Q_{1.1,2}(t,\,x,\,y,\,z)|$ in Eq.(34) for $\epsilon _0=2,\,\epsilon _1=3, \,\epsilon _2=1, \,\alpha _1=0.6,\, \alpha _2=0.7,\,\beta _1=0.8,\,\beta _2=0.9,\,\beta _3=0.7,\,\varepsilon =0,\,a_1= 0.8,\,a_2=0.9,$ and $\,a_3=0.7$, **(a)** The dynamics of singular soliton $Q_{1.1,2}(t,\,x,\,y,\,1)$ with time *t* in 3D, **(b)** The dynamics of singular soliton $Q_{1.1,2}(t,\,x,\,0.5,\,1)$ with time *t* in 2D, **(c), (d),** and **(e)** The density plots of $|Q_{1.1,2}(0,\,x,\,y,\,1)|$, $|Q_{1.1,2}(3,\,x,\,y,\,1)|$, and $|Q_{1.1,2}(5,\,x,\,y,\,1)|$, respectively.

**Fig. 3:**
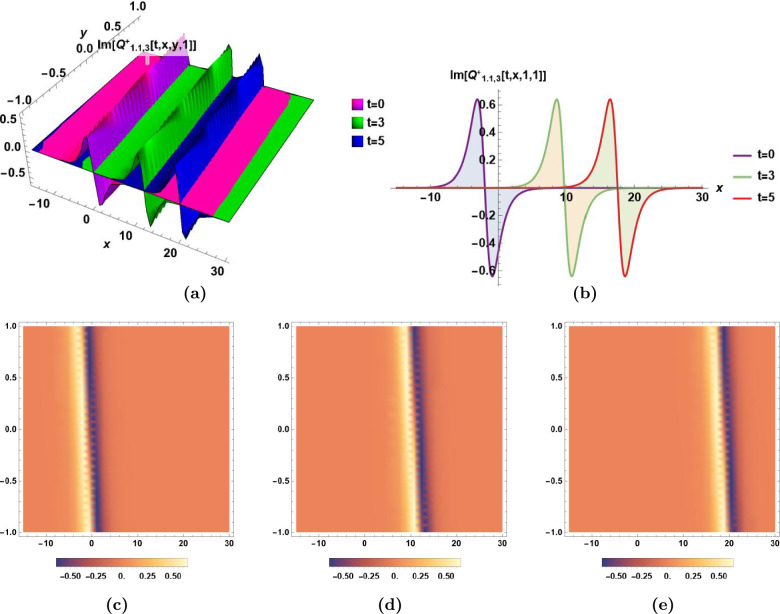
Combo bright-dark soliton $Im[Q_{1.1,3}^{+}(t,\,x,\,y,\,z)]$ in Eq.(35) for $\epsilon _0=2,\,\epsilon _1=3, \,\epsilon _2=1,\, \alpha _1=0.6,\,\alpha _2=0.7,\,\beta _1=0.8,\,\beta _2=0.9,\,\beta _3=0.7,\,\varepsilon =0,\,a_1= 0.8,\,a_2=0.9,$ and $\,a_3=0.7$, **(a)** The dynamics of combo bright-dark soliton $Im[Q_{1.1,3}^{+}(t,\,x,\,y,\,1)]$ with time *t* in 3D, **(b)** The dynamics of combo bright-dark soliton $Im[Q_{1.1,3}^{+}(t,\,x,\,1,\,1)]$ with time *t* in 2D, **(c), (d),** and** (e)** The density plots of $Im[Q_{1.1,3}^{+}(0,\,x,\,y,\,1)]$, $Im[Q_{1.1,3}^{+}(3,\,x,\,y,\,1)]$, and $Im[Q_{1.1,3}^{+}(5,\,x,\,y,\,1)]$, respectively.

**Fig. 4:**
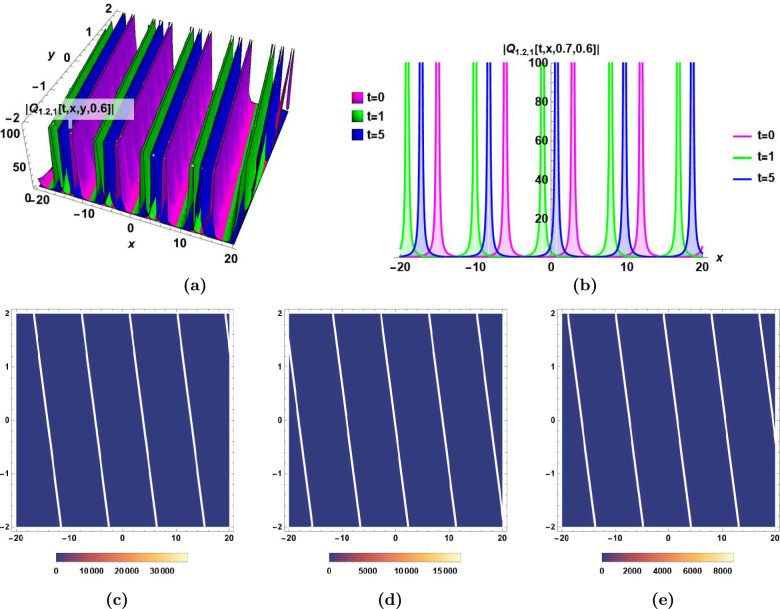
Singular periodic solution $|Q_{1.2,1}(t,\,x,\,y,\,z)|$ in Eq.(40) for $\epsilon _0=0.5,\,\epsilon _1=1, \,\epsilon _2=1,\, \alpha _1=0.6,\,\alpha _2=0.8,\,\beta _1=0.9,\,\beta _2=0.7,\,\beta _3=0.6,\,\varepsilon =0,\,a_1= 0.7,\,a_2=0.9,$ and $\,a_3=0.8$, **(a)** The dynamics of combo bright-dark soliton $|Q_{1.2,1}(t,\,x,\,y,\,0.6)|$ with time *t* in 3D, **(b)** The dynamics of combo bright-dark soliton $|Q_{1.2,1}(t,\,x,\,0.7,\,0.6)|$ with time *t* in 2D, **(c), (d),** and** (e)** The density plots of $|Q_{1.2,1}(0,\,x,\,y,\,0.6)|$, $|Q_{1.2,1}(1,\,x,\,y,\,0.6)|$, and $|Q_{1.2,1}(5,\,x,\,y,\,0.6)|$, respectively.

**Fig. 5:**
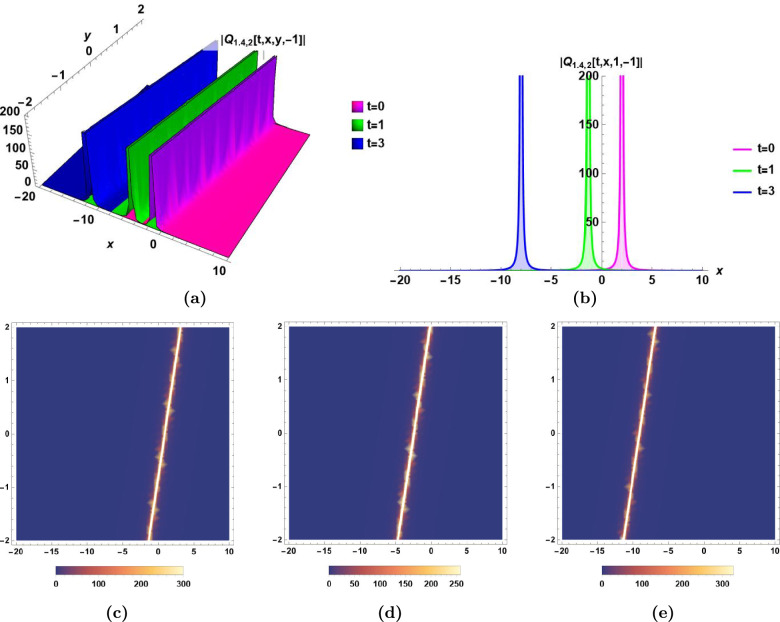
Rational solution $|Q_{1.4,2}(t,\,x,\,y,\,z)|$ in Eq.(50) for $\epsilon _0=-1,\,\epsilon _1=-0.6, \,\alpha _1=0.6, \,\alpha _2=0.7,\,\beta _1=1.5,\,\beta _2=1,\,\beta _3=-2,\,\varepsilon =0,\,a_1=- 0.8,\,a_2=0.9,$ and $\,a_3=-0.7$, **(a)** The dynamics of rational solution $|Q_{1.4,2}(t,\,x,\,y,\,-1)|$ with time *t* in 3D, **(b)** The dynamics of rational solution $|Q_{1.4,2}(t,\,x,\,1,\,-1)|$ with time *t* in 2D, **(c), (d),** and** (e)** The density plots of $|Q_{1.4,2}(0,\,x,\,y,\,-1)|$, $|Q_{1.4,2}(1,\,x,\,y,\,-1)|$, and $|Q_{1.4,2}(3,\,x,\,y,\,-1)|$, respectively.

**Fig. 6:**
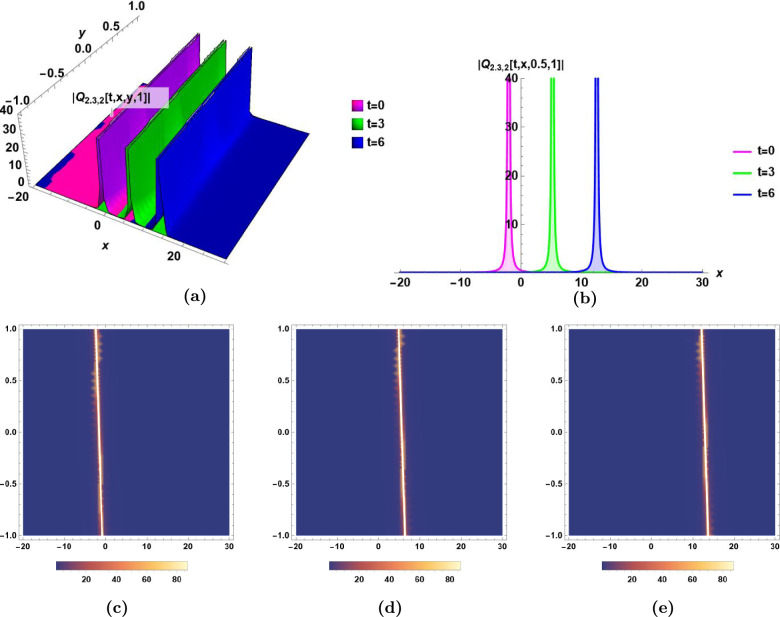
Exponential solution $|Q_{2.3,2}(t,\,x,\,y,\,z)|$ in Eq.(67) for $\,\epsilon _0=0,\,\epsilon _1=-0.6,\,\epsilon _2=1,\,v=-1.5, \,\alpha _1=0.9,\,\alpha _2=0.8,\,\beta _1=0.5,\,\beta _2=0.6,\,\beta _3=0.7,\,\varepsilon =0,\,a_1= 0.9,\,a_2=0.7,$ and $\,a_3=0.8$. **(a)** The dynamics of exponential solution $|Q_{2.3,2}(t,\,x,\,y,\,1)|$ with time *t* in 3D, **(b)** The dynamics of exponential solution $|Q_{2.3,2}(t,\,x,\,0.5,\,1)|$ with time *t* in 2D, **(c), (d),** and **(e)** The density plots of $|Q_{2.3,2}(0,\,x,\,y,\,1)|$, $|Q_{2.3,2}(3,\,x,\,y,\,1)|$, and $|Q_{2.3,2}(6,\,x,\,y,\,1)|$, respectively.

In the following, the dynamics of some derived solutions are graphically illustrated in Fig.1–6 with 2-dimensional, 3-dimensional and density plots, and the descriptions of their different characteristics are presented.

- Bright soliton:
  - The dynamics of bright soliton $Q_{1.1,1}(t,\,x,\,y,\,z)$ in Eq.(33) with time are visualized by 2-dimensional, 3-dimensional, and density plots in Fig.1 for $t=0,\,t=1,\,$ and $t=3$.
  - The main characteristics of bright soliton are the maximum intensity centered peak, and the propagation for long distances without distortion. This solution is essential in optical communication systems due to its propagation for long distances without distortion. The 2-dimensional, and 3-dimensional emphasis the stability of propagation of bright soliton which maintains the integrity of the signal, and enables the high-performance in optical networks^43,45^.
- Singular soliton
  - The dynamics of singular soliton $|Q_{1.1,2}(t,\,x,\,y,\,z)|$ in Eq.(34) with time are visualized by 2-dimensional, 3-dimensional, and density plots in Fig.2 for $t=0,\,t=3,\,$ and $t=5$.
  - The main characteristic of singular soliton is the infinite centered amplitude. This huge sudden change in amplitude is important in studying extreme waves to help developing realistic models^5,43,44^.
- Combo bright-dark soliton
  - The dynamics of combo bright-dark soliton $Im[Q_{1.1,3}(t,\,x,\,y,\,z)]$ in Eq.(35) with time are visualized by 2-dimensional, 3-dimensional, and density plots in Fig.3 for $t=0,\,t=3,\,$ and $t=5$.
  - The main characteristic of combo bright-dark soliton is the presence of a wave packet that combines both the dark and bright solitons. The bright soliton presents a peak of the wave amplitude intensity while the dark soliton presents a dip of the wave amplitude intensity, both coexisting within the same wave. The stability of this soliton is evident as they remain stable over long distances.^47^
- Singular periodic solution
  - The dynamics of singular periodic solution $|Q_{1.2,1}(t,\,x,\,y,\,z)|$ in Eq.(40) with time are visualized by 2-dimensional, 3-dimensional, and density plots in Fig.4 for $t=0,\,t=1,\,$ and $t=5$.
  - The main characteristic of the singular periodic solution is the oscillatory nature which has many usages in advanced signal processing techniques. The oscillatory nature of this solution is used for wavelength conversion, and other signal processing applications in optical communication systems^5,45^.
- Rational solution
  - The dynamics of rational solution $|Q_{1.4,2}(t,\,x,\,y,\,z)|$ in Eq.(50) with time are visualized by 2-dimensional, 3-dimensional, and density plots in Fig.5 for $t=0,\,t=1,\,$ and $t=3$.
  - The main characteristic of the rational solution is the sudden huge change in the wave’s amplitude. It is used to model the unexpected nonlinear wave phenomena in ocean called rogue waves^48^. The graphical illustration of the rational solution shows its potential applications in the modeling of interactions of complex waves, and in the optimization of signal transmission^43^.
- Exponential solution
  - The dynamics of exponential solution $|Q_{2.3,2}(t,\,x,\,y,\,z)|$ in Eq.(67) with time are visualized by 2-dimensional, 3-dimensional, and density plots in Fig.6 for $t=0,\,t=3,\,$ and $t=6$.
  - The main characteristic of the exponential solution is the rapid initial growth (or decay) in the wave amplitude at a rate proportional to the wave’s current size. This solution plays important role in studying systems with turbulence^43,46^. The exponential growth in ocean waves is driven by wind forcing and nonlinear interactions. The study of exponential growth is essential to understand how wind generates and builds waves from an initially calm water surface^49,50^.

### Graphical illustration of MI analysis

**Fig. 7:**
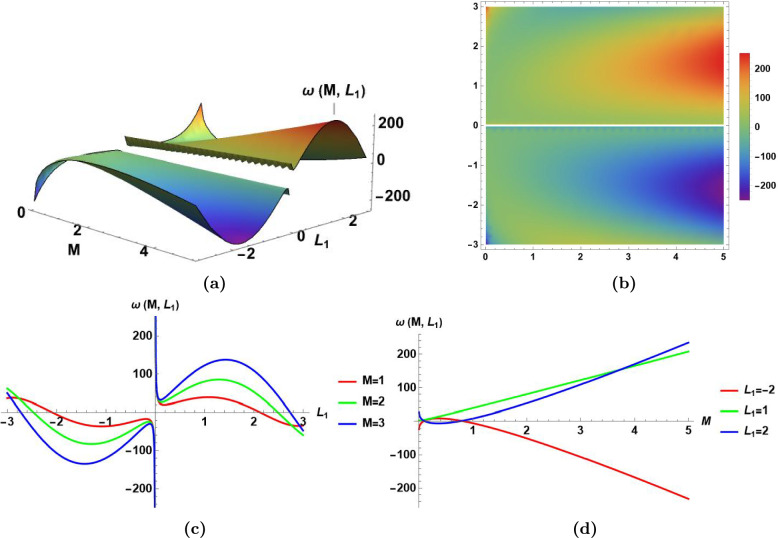
The perturbation frequency $$\omega ({\mathcal {M}}$$,$$L_1)$$ in Eq.(76) for $$\alpha _1=0.8,\,\alpha _2=0.8, \,\beta _1=0.9, \, \beta _2=0.7, \, \beta _3=0.6,\,L_2=0.8,\,$$ and $$L_3=0.9$$, **(a)** The visualization of the perturbation frequency $$\omega ({\mathcal {M}}$$,$$L_1)$$ as a function in the perturbation wave number $$L_1$$, and the normalized power $${\mathcal {M}}$$ in 3D, **(b)** The density plot of the perturbation frequency $$\omega ({\mathcal {M}}$$,$$L_1)$$, **(c)** The 2D visualization of the perturbation frequency $$\omega ({\mathcal {M}}$$,$$L_1)$$ versus the perturbation wave number $$L_1$$ while changing the value of the normalized power $${\mathcal {M}}$$, **(d)** The 2D visualization of the perturbation frequency $$\omega ({\mathcal {M}}$$,$$L_1)$$ versus the normalized power $${\mathcal {M}}$$ while changing the value of the perturbation wave number $$L_1$$,

**Fig. 8:**
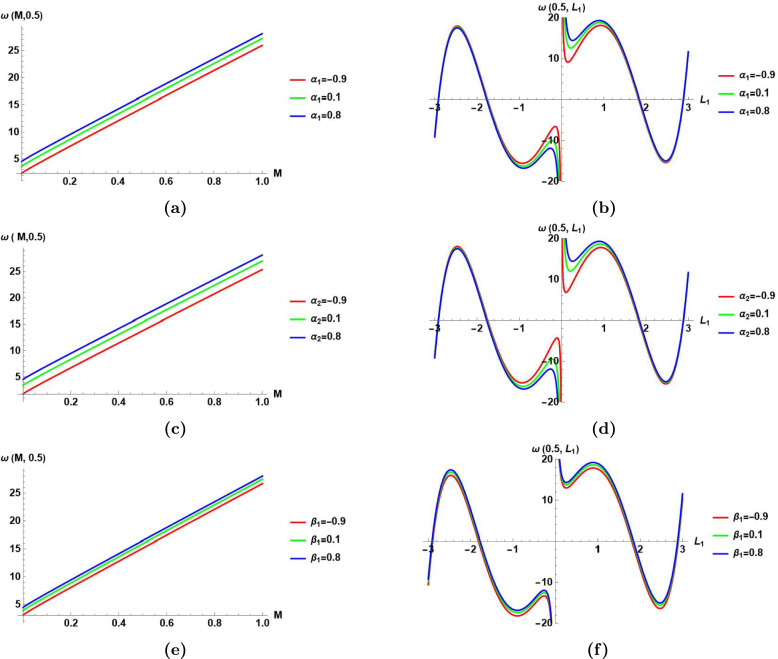
The effect of changing the coefficients $$\alpha _{1}, \alpha _{2},$$ and $$\beta _{1}$$ in Eq.(1) on the perturbation frequency $$\omega ({\mathcal {M}}$$,$$L_1)$$ in Eq.(76), **(a)-(b)** The effect of changing $$\alpha _{1}$$ on the perturbation frequency $$\omega ({\mathcal {M}}$$,$$L_1)$$ versus the normalized power with $$L_1=0.5$$ in **(a)**, and versus the perturbation wave number $$L_1$$ with $${\mathcal {M}}$$ $$=0.5$$ in **(b)** for $$\alpha _2=0.8, \,\beta _1=0.9, \, \beta _2=0.7, \, \beta _3=0.6,\,L_2=0.8,\,$$ and $$L_3=0.9$$, **(c)-(d)** The effect of changing $$\alpha _{2}$$ on the perturbation frequency $$\omega ({\mathcal {M}}$$,$$L_1)$$ versus the normalized power with $$L_1=0.5$$ in **(c)**, and versus the perturbation wave number $$L_1$$ with $${\mathcal {M}}$$ $$=0.5$$ in **(d)** for $$\alpha _1=0.8, \,\beta _1=0.9, \, \beta _2=0.7, \, \beta _3=0.6,\,L_2=0.8,\,$$ and $$L_3=0.9$$, **(e)-(f)** The effect of changing $$\beta _{1}$$ on the perturbation frequency $$\omega ({\mathcal {M}}$$,$$L_1)$$ versus the normalized power with $$L_1=0.5$$ in **(e)**, and versus the perturbation wave number $$L_1$$ with $${\mathcal {M}}$$ $$=0.5$$ in **(f)** for $$\alpha _1=0.8, \,\alpha _2=0.8, \, \beta _2=0.7, \, \beta _3=0.6,\,L_2=0.8,\,$$ and $$L_3=0.9$$.

**Fig. 9:**
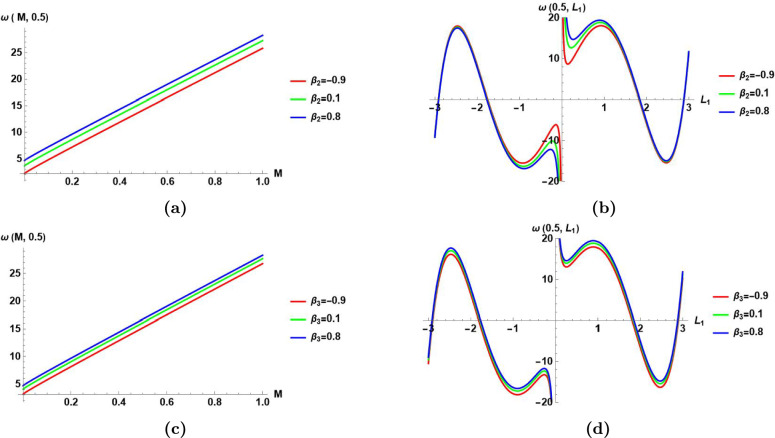
The effect of changing the coefficients $$\beta _{2},$$ and $$\beta _{3}$$ in Eq.(1) on the perturbation frequency $$\omega ({\mathcal {M}}$$,$$L_1)$$ in Eq.(76), **(a)-(b)** The effect of changing $$\beta _{2}$$ on the perturbation frequency $$\omega ({\mathcal {M}}$$,$$L_1)$$ versus the normalized power with $$L_1=0.5$$ in **(a)**, and versus the perturbation wave number $$L_1$$ with $${\mathcal {M}}$$ $$=0.5$$ in **(b)** for $$\alpha _1=0.8, \,\alpha _2=0.8, \, \beta _1=0.9, \, \beta _3=0.6,\,L_2=0.8,\,$$ and $$L_3=0.9$$, **(c)-(d)** The effect of changing $$\beta _{3}$$ on the perturbation frequency $$\omega ({\mathcal {M}}$$,$$L_1)$$ versus the normalized power with $$L_1=0.5$$ in **(c)**, and versus the perturbation wave number $$L_1$$ with $${\mathcal {M}}$$ $$=0.5$$ in **(d)** for $$\alpha _2=0.8, \,\beta _1=0.9, \, \beta _2=0.7, \, \alpha _1=0.8,\,L_2=0.8,\,$$ and $$L_3=0.9$$.

**Fig. 10:**
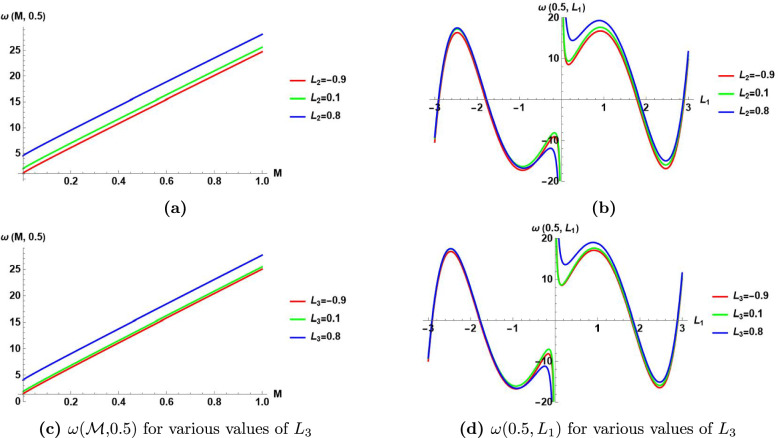
The effect of changing the perturbation wave numbers $L_{2},$ and $L_{3}$ in Eq.(73) on the perturbation frequency $\omega ({\mathcal {M}},L_1)$ in Eq.(76), **(a)-(b)** The effect of changing $L_{2}$ on the perturbation frequency $\omega ({\mathcal {M}},L_1)$ versus the normalized power with $L_1=0.5$ in **(a)**, and versus the perturbation wave number $L_1$ with ${\mathcal {M}}=0.5$ in **(b)** for $\alpha _1=0.8, \,\beta _1=0.9, \, \beta _2=0.7, \, \beta _3=0.6,\,\alpha _2=0.8,\,$ and $L_3=0.9$, **(c)-(d)** The effect of changing $L_{3}$ on the perturbation frequency $\omega ({\mathcal {M}},L_1)$ versus the normalized power with $L_1=0.5$ in **(c)**, and versus the perturbation wave number $L_1$ with ${\mathcal {M}}=0.5$ in **(d)** for $\alpha _1=0.8, \,\alpha _2=0.8, \, \beta _2=0.7, \, \beta _3=0.6,\,L_2=0.8,\,$ and $\beta _1=0.9$.

This subsection presents the visualization of the obtained results of MI analysis in sect. 4 by the aid of Fig.7–10.

- Fig. 7

- Presents the perturbation frequency $\omega ({\mathcal {M}},$ $\,L_{1})$ derived in Eq.(76) as a function in the normalized power ${\mathcal {M}}$, and the perturbation wave number $L_1$ in 3D.

- It shows a nonlinearity behavior, and singularity of the perturbation frequency at $L_1=0$.
- It shows that the increase of the normalized power value induces a maxima for positive value of wave number $L_1$, and a minima for negative value of wave number $L_1$.

(b) Presents the density plot of the perturbation frequency $\omega ({\mathcal {M}},$ $\,L_{1}$).
(c) Presents the perturbation frequency $\omega ({\mathcal {M}},\,L_{1})$ as a function in $L_1$ only for three different values of the normalized power ${\mathcal {M}}=\{1,2,3\}$.

- It shows that the perturbation frequency has an asymptotic line at $L_1=0$.
- It shows that, at constant value of normalized power ${\mathcal {M}}$, the perturbation frequency curve is concave down for positive values of $L_1$, and concave up for negative values of $L_1$.
- It shows that as the normalized power value increases, the convexity of frequency curve, and the absolute value of maxima and minima increase.

(d) Presents perturbation frequency $\omega ({\mathcal {M}},\,L_{1})$ as a function in the normalized power ${\mathcal {M}}$ only for three different values of the wave number $L_1=\{-2,1,2\}$.

- It shows that the perturbation frequency has a direct relationship with the normalized power ${\mathcal {M}}$ at fixed value of wave number $L_1$.

- Fig.8

- Shows the perturbation frequency $\omega ({\mathcal {M}}$,0.5) in Eq.(76) versus the normalized power ${\mathcal {M}}$ for three different values of $\alpha _{1}=\{-0.9,0.1,0.8\}$.

- It shows that the perturbation frequency has a linear relationship with the normalized power ${\mathcal {M}}$ for constant value of wave number $L_1$, and a constant value of $\alpha _1$.
- It shows that at any selected value of the normalized power ${\mathcal {M}}$, the increase of $\alpha _1$ increases the perturbation frequency.

(b) Shows the perturbation frequency $\omega (0.5,L_1)$ in Eq.(76) versus the wave number $L_1$ for three different values of $\alpha _{1}=\{-0.9,0.1,0.8\}$.

- It shows that the perturbation frequency has a nonlinear relationship with the wave number $L_1$ for constant value of the normalized power ${\mathcal {M}}$, and a constant value of $\alpha _1$.
- It shows that at small values of the wave number $L_1$, the increase of $\alpha _1$ increases the perturbation frequency. But, at higher values of the wave number $L_1$, the increase in the value of $\alpha _1$ does not significantly affect the value of the perturbation frequency.

(c) Visualizes $\omega ({\mathcal {M}}$,0.5) in Eq.(76) versus the normalized power ${\mathcal {M}}$ for three different values of $\alpha _{2}=\{-0.9,0.1,0.8\}$.

- It shows that the perturbation frequency has a linear relationship with the normalized power ${\mathcal {M}}$ for constant value of wave number $L_1$, and a constant value of $\alpha _2$.
- It shows that at any selected value of the normalized power ${\mathcal {M}}$, the increase of $\alpha _2$ increases the perturbation frequency.

(d) Visualizes $\omega (0.5,L_1)$ versus the wave number $L_1$ for three different values of $\alpha _{2}=\{-0.9,0.1,0.8\}$.

- It shows that the perturbation frequency has a nonlinear relationship with the wave number $L_1$ for constant value of the normalized power ${\mathcal {M}}$, and a constant value of $\alpha _2$.
- It shows that at small values of the wave number $L_1$, the increase of $\alpha _2$ increases the perturbation frequency. But, at higher values of the wave number $L_1$, the increase in the value of $\alpha _2$ does not significantly affect the value of the perturbation frequency.

(e) Visualizes the perturbation frequency $\omega ({\mathcal {M}}$,0.5) in Eq.(76) versus ${\mathcal {M}}$ for three different values of $\beta _{1}=\{-0.9,0.1,0.8\}$.

- It shows that the perturbation frequency has a linear relationship with the normalized power ${\mathcal {M}}$ for constant value of wave number $L_1$, and a constant value of $\beta _1$.
- It shows that at any selected value of the normalized power ${\mathcal {M}}$, the increase of $\beta _1$ increases the perturbation frequency.

(f) Visualizes the perturbation frequency $\omega (0.5,L_1)$ in Eq.(76) versus $L_1$ for three different values of $\beta _{1}=\{-0.9,0.1,0.8\}$.

- It shows that the perturbation frequency has a nonlinear relationship with the wave number $L_1$ for constant value of the normalized power ${\mathcal {M}}$, and a constant value of $\beta _{1}$.
- It shows that the effect of changing $\beta _{1}$ on the perturbation frequency is very small at any value of the wave number $L_1$.

- Fig.9

- Visualizes the perturbation frequency $\omega ({\mathcal {M}}$,0.5) in Eq.(76) versus ${\mathcal {M}}$ for three different values of $\beta _{2}=\{-0.9,0.1,0.8\}$.

- It shows that the frequency perturbation has a linear relationship with the normalized power ${\mathcal {M}}$ for constant value of wave number $L_1$, and a constant value of $\beta _2$.
- It shows that at any selected value of the normalized power ${\mathcal {M}}$, the increase of $\beta _2$ increases the perturbation frequency.

(b) Visualizes $\omega (0.5,L_1)$ versus $L_1$ for three different values of $\beta _{2}=\{-0.9,0.1,0.8\}$.

- It shows that the perturbation frequency has a nonlinear relationship with the wave number $L_1$ for constant value of the normalized power ${\mathcal {M}}$, and a constant value of $\beta _{2}$.
- It shows that for small values of the wave number $L_1$, the increase of the value of $\beta _{2}$ increases the perturbation frequency. But, for higher values of the wave number $L_1$, the increase in the value of $\beta _2$ does not significantly affect the value of the perturbation frequency.

(c) Visualizes the perturbation frequency $\omega ({\mathcal {M}}$,0.5) in Eq.(76) versus ${\mathcal {M}}$ for three different values of $\beta _{3}=\{-0.9,0.1,0.8\}$.

- It shows that the perturbation frequency has a linear relationship with the normalized power ${\mathcal {M}}$ for constant value of wave number $L_1$, and a constant value of $\beta _3$.
- It shows that at any selected value of the normalized power ${\mathcal {M}}$, the increase of $\beta _3$ increases the perturbation frequency.

(d) Visualizes the perturbation frequency $\omega (0.5,L_1)$ versus the wave number $L_1$ for three different values of $\beta _{3}=\{-0.9,0.1,0.8\}$.

- It shows that the perturbation frequency has a nonlinear relationship with the wave number $L_1$ for constant value of the normalized power ${\mathcal {M}}$, and a constant value of $\beta _{3}$.
- It shows that the effect of changing $\beta _{3}$ on the perturbation frequency is very small at any value of the wave number $L_1$.

- Fig.10

- Visualizes the perturbation frequency $\omega ({\mathcal {M}}$,0.5) in Eq.(76) versus the normalized power ${\mathcal {M}}$ for three different values of $L_2=\{-0.9,0.1,0.8\}$.

- It shows that the perturbation frequency has a linear relationship with the normalized power ${\mathcal {M}}$ for constant value of wave number $L_1$, and a constant value of $L_2$.
- It shows that at any selected value of the normalized power ${\mathcal {M}}$, the increase of $L_2$ increases the perturbation frequency.

(b) Visualizes the perturbation frequency $\omega (0.5,L_1)$ in Eq.(76) versus the wave number $L_1$ for three different values of $L_{2}=\{-0.9,0.1,0.8\}$.

- It shows that the frequency perturbation has a nonlinear relationship with the wave number $L_1$ for constant value of the normalized power ${\mathcal {M}}$, and a constant value of $L_2$.
- It shows that the effect of changing $L_2$ on the perturbation frequency is significant at different values of the wave number $L_1$.

(c) Visualizes the perturbation frequency $\omega ({\mathcal {M}}$,0.5) in Eq.(76) versus the normalized power ${\mathcal {M}}$ for three different values of $L_{3}=\{-0.9,0.1,0.8\}$.

- It shows that the perturbation frequency has a linear relationship with the normalized power ${\mathcal {M}}$ for constant value of wave number $L_1$, and a constant value of $L_3$.
- It shows that at any selected value of the normalized power ${\mathcal {M}}$, the increase of $L_3$ increases the perturbation frequency.

(d) Visualizes the perturbation frequency $\omega (0.5,L_1)$ in Eq.(76) versus the wave number $L_1$ for three different values of $L_{3}=\{-0.9,0.1,0.8\}$.

- It shows that the perturbation frequency has a nonlinear relationship with the wave number $L_1$ for constant value of the normalized power ${\mathcal {M}}$, and a constant value of $L_{3}$.
- It shows that the effect of changing $L_{3}$ on the perturbation frequency is significant at different values of the wave number $L_1$.

## Conclusion

The current work derived new analytic solutions for the (3+1)-dimensional Kadomtsev–Petviashvili– Sawada–Kotera–Ramani equation (KP-SKRE) in Eq.(1) by the novel application of the improved generalized Riccati equation mapping method (IGREMM). The primary application of Eq.(1) is in the modeling of shallow water waves. But it is used in other applications like, for example, plasma physics where it describes the wave propagation in plasma, which is a matter of ions and electrons. Another example of applications is oceanography, where it describes ocean waves and is especially important to study rogue waves, and many other applications as nonlinear optics, fluid mechanics, and electrical engineering^26^. According to our knowledge, previous obtained results in the literature included multiple-soliton, and lump solutions in^26^by simplified Hirota’s scheme, an exponential rational function solution in^35^ by the Kudryashov method, single and combined Jacobi elliptic function solutions in^36^ by the Jacobi elliptic function expansion, and hybrid solutions in^37^ by the Hirota bilinear method. The current work introduced abundant novel analytic solutions for the (3+1)-dimensional KP-SKRE in Eq.(1). The IGREMM succeeded to achieve different novel forms of exact solutions as bright, singular, combo bright-dark solitons in addition to hyperbolic, singular periodic, rational, and exponential solutions. Derived solutions, in current work, were substituted in Eq.(1) and were found to satisfy it. The Bäcklund transformation of IGREMM in Eq.(26) can be used to construct additional novel forms of solution. Also, in current work, MI analysis for Eq.(1) is derived by linear stability analysis. MI analysis showed the stability of Eq.(1) under the effect of small perturbations. This is due to the real value of frequency of perturbation which prevents any exponential growth of small perturbation and hence the introduced perturbation decays to zero. Using suitable parameters’ values satisfying the restricting conditions, the 2-dimensional, 3-dimensional, and density plots were presented to graphically illustrate the dynamics of some derived analytic solutions. The 2-dimensional, and 3- dimensional plots of MI analysis outcomes were also presented in this work. The future work of this study can consider the study of the impact of parameter selection on the model’s solutions. Furthermore, applying new analytic methods to find dark soliton and other new solutions for Eq.(1) that were not reported before.

## Data Availability

The datasets used and/or analysed during the current study are available from the corresponding author on reasonable request.
